# Phenylhydrazone and Quinazoline Derivatives from the Cold-Seep-Derived Fungus *Penicillium oxalicum*

**DOI:** 10.3390/md19010009

**Published:** 2020-12-28

**Authors:** Ya-Ping Liu, Sheng-Tao Fang, Zhen-Zhen Shi, Bin-Gui Wang, Xiao-Nian Li, Nai-Yun Ji

**Affiliations:** 1 Yantai Institute of Coastal Zone Research, Center for Ocean Mega-Science, Chinese Academy of Sciences, Yantai 264003, China; ypliu@yic.ac.cn (Y.-P.L.); stfang@yic.ac.cn (S.-T.F.); zzshi@yic.ac.cn (Z.-Z.S.); 2 University of Chinese Academy of Sciences, Beijing 100049, China; 3 Laboratory of Marine Biology and Biotechnology of the Qingdao National Laboratory for Marine Science and Technology, Key Laboratory of Experimental Marine Biology at the Institute of Oceanology, Center for Ocean Mega-Science, Chinese Academy of Sciences, Qingdao 266071, China; wangbg@ms.qdio.ac.cn; 4 Kunming Institute of Botany, Chinese Academy of Sciences, Kunming 650201, China; lixiaonian@mail.kib.ac.cn

**Keywords:** *Penicillium oxalicum*, deep sea, cold seep, phenylhydrazone, quinazoline

## Abstract

Three new phenylhydrazones, penoxahydrazones A–C (compounds **1**–**3**), and two new quinazolines, penoxazolones A (compound **4**) and B (compound **5**), with unique linkages were isolated from the fungus *Penicillium oxalicum* obtained from the deep sea cold seep. Their structures and relative configurations were assigned by analysis of 1D/2D NMR and mass spectroscopic data, and the absolute configurations of **1**, **4**, and **5** were established on the basis of X-ray crystallography or ECD calculations. Compound **1** represents the first natural phenylhydrazone-bearing steroid, while compounds **2** and **3** are rarely occurring phenylhydrazone tautomers. Compounds **4** and **5** are enantiomers that feature quinazoline and cinnamic acid units. Some isolates exhibited inhibition of several marine phytoplankton species and marine-derived bacteria.

## 1. Introduction

Microorganisms of different origin may possess unique genomes and potentials that enable them to produce rare metabolites [1,2]. As an important class of microorganisms, fungi are substantial sources of striking secondary metabolites with diverse structures and bioactivities. The fungal species *Penicillium oxalicum* is widespread in terrestrial and marine environments, and its secondary metabolites are abundant. In the investigation towards terrestrial-derived *P. oxalicum*, an azo compound [3], a diterpene [4], a diphenylmethanone [5], a spiro-oxindole alkaloid [6], isochroman carboxylic acids [3], and polyketides [7] were obtained from soil-derived isolates, several limonoids [8], butyrolactones [9], and isocoumarins [10] were discovered from plant-derived isolates, and alkaloids with 1,3-thiazole and 1,2,4-thiadiazole units [11] were separated from an animal-derived isolate. On the other hand, more and more marine-derived *P. oxalicum* strains were chemically examined. A variety of metabolites including chromones [12,13,14,15] from marine-animal-derived strains, phenolic enamides [16], meroterpenoids [16], and alkaloids [17,18] from marine-plant-derived strains, and secalonic acids [19,20,21], anthraquinones [22], alkaloids [23], and a diphenylmethanone [21] from marine-sediment-derived strains were characterized. It is obvious that the metabolic profiles of *P. oxalicum* strains varied with their habitats. Secondary metabolite biosynthesis genes from fungi have been found to be expressed in deep sea sediments [24,25]. However, chemical surveys were rarely performed on deep-sea-derived *P. oxalicum* strains, especially cold-seep-derived ones.

## 2. Results and Discussion

**Figure 1:**
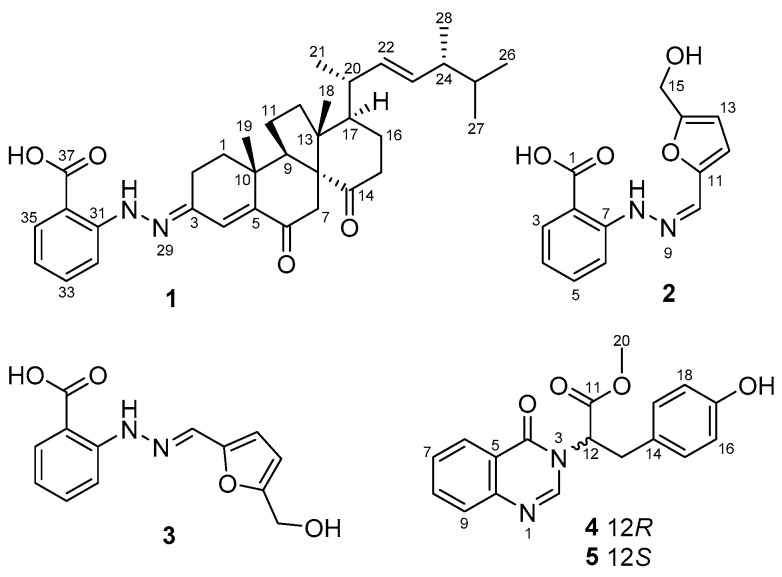
Chemical structures of compounds **1**–**5**.

*Penicillium oxalicum* 13–37 was isolated from the deep sea cold seep sediments. After static fermentation at room temperature for 30 days, the cultures were extracted with organic solvents and then purified by repeated column chromatography on silica gel, RP-18, and Sephadex LH-20 as well as semipreparative HPLC to yield penoxahydrazones A–C (**1**–**3**), penoxazolones A (**4**) and B (**5**), and dankasterone A (Figure 1). The structures of these compounds were identified by extensive 1D/2D NMR and mass spectrometric data, X-ray crystallographic analysis or ECD calculations.

**Figure 2:**
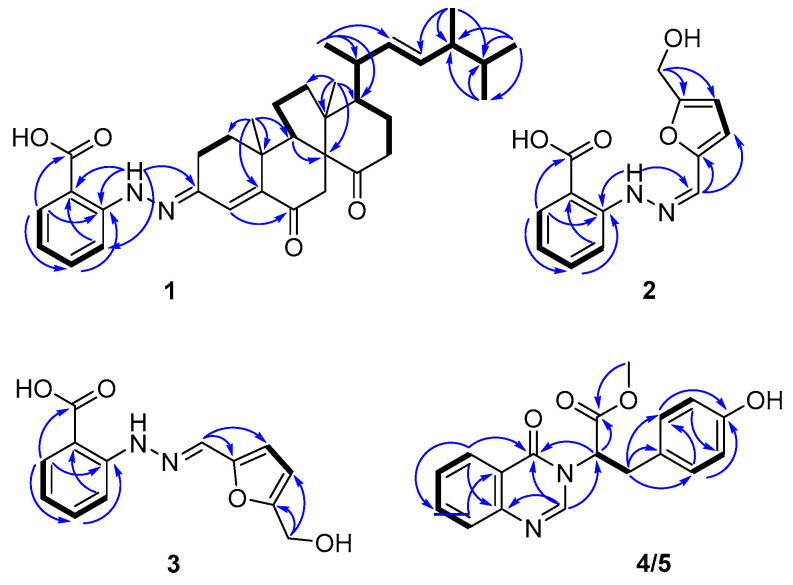
Key COSY (bold lines) and HMBC (arrows) correlations of compounds **1**–**5**.

**Figure 3:**
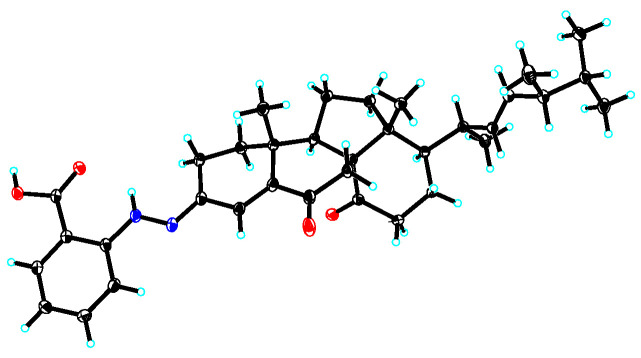
X-ray crystallographic structure of compound **1**.

Penoxahydrazone A (**1**) was purified as yellow crystals. The molecular formula C_35_H_46_N_2_O_4_ was determined by interpretation of HRESIMS data. In combination with HSQC correlations, the ^1^H NMR spectrum (Table 1) displayed four methyl doublets, two methyl singlets, two double doublets and one singlet ascribable to three olefinic protons, two double doublets and two doublets attributable to four aromatic protons, and a range of signals at δ_H_ 1.3–2.9 for seven methylenes and five methines. The ^13^C NMR spectrum (Table 1) exhibited 35 resonances, classified into six methyls, seven methylenes, twelve methines, and ten non-protonated carbons by DEPT experiments. The above NMR data partially resembled those for co-isolated dankasterone A [26]. Replacing the signal at δ_C_ 199.1 for the C-3 carbonyl group in dankasterone A, a signal at δ_C_ 144.4 appeared in the ^13^C NMR spectrum of **1**, and this carbon atom was located at C-3 by its heteronuclear multiple bond correlation (HMBC)with H-1a. Additionally, the remaining NMR data corresponded to an ortho-substituted benzoic acid unit [27], as supported by the ^1^H-^1^H chemical shift correlation spectroscopy (COSY) correlations from H-32 thoroughly to H-35 and the HMBC correlations from H-32 to C-36, from H-33 to C-31 and C-35, and from H-35 to C-33 and C-37. To satisfy the elemental composition, an azo unit was situated between C-3 and C-31, which was supported by the HMBC correlations from H-30 to C-3, C-31, C-32, and C-36. Thus, the whole connectivity of **1** was established, which was further validated by other COSY and HMBC correlations (Figure 2). The absolute configuration was determined by X-ray crystallographic analysis (Figure 3). A suitable single crystal was obtained from a solution of EtOH with a drop of water and then subjected to the X-ray diffraction analysis using Cu Kα radiation [28]. Consequently, the absolute configuration of **1** was assigned to be 8R, 9R, 10R, 13R, 17R, 20R, and 24R. Although more than 200 natural molecules with a nitrogen–nitrogen bond have been found so far, phenylhydrazone derivatives rarely occurred [27,29]. Furthermore, **1** represents the first natural phenylhydrazone-bearing steroid.

Penoxahydrazones B(**2**) and C(**3**) were obtained as a brown powder. During the purification process, **2** could gradually turn to **3** and vice versa. The sodium adducts ion peaks at *m*/*z* 283.0695 and *m*/*z* 283.0691 determined by high performance liquid chromatography-electrospray mass spectrometry (HPLC-ESIMS) suggested that **2** and **3** feature the same molecular formula C_13_H_12_N_2_O_4_. On the basis of these characteristics, it is inferred that these two compounds should be a pair of tautomers. The ^1^H and ^13^C NMR spectra (Table 2) showed two sets of signals with a ratio of 2:1. Aided by HSQC data, they indicated the presence of one oxymethylene, 11 aromatic/olefinic methines, and one carboxyl in each compound. Similar to the analysis of the structure of compound **1**, an ortho-substituted benzoic acid unit was deduced to be present in both **2** and **3** based on their NMR data [27], which was further supported by the COSY and the HMBC correlations (Figure 2). Compared to the NMR data of δ-hydroxymethyl-α-vinylfuran [30], the remaining carbon signals could be assigned to δ-hydroxymethyl furan attached by an olefinic methine group at α position, which was verified by the COSY correlation between H-12 and H-13 and the HMBC correlations from H-10 to C-11 and C-12 and from H_2_-15 to C-13 and C-14. To match the molecular formula, these two moieties were linked via an azo unit, as seen from the HMBC correlation from the de-shielded exchangeable proton to C-7 and C-10 in **2**. Although this HMBC correlation was not detected for **3**, the similarities of NMR data for C-7 and C-10 between **2** and **3** suggested the same connectivity of them. Through analysis of the whole structures of **2** and **3**, their tautomerization probably arose from the geometric isomerization of the double bond between N-9 and C-10. The ^13^C NMR data of 9*Z* and 9*E* isomers were computed using the gauge-independent atomic orbital (GIAO) method at the B3LYP/6-31+G(d,p) level via Gaussian 09 software [31] and then were input into Sarotti’s DP4+ sheet (https://sarotti-nmr.weebly.com) [32]. According to the DP4+ probabilities for ^1^H (100% between 9*Z* isomer and **2**, 100% between 9E isomer and **3**, Tables S1 and S2) and ^13^C NMR data (99.99% between 9*Z* isomer and **2**, 98.80% between 9*E* isomer and **3**, Tables S3 and S4), **2** and **3** were proposed to possess 9*Z* and 9*E* configurations, respectively. These two tautomers are possibly formed by the reaction between 2-hydrazinylbenzoic acid and 5-hydroxymethylfurfural, and the latter is a valuable platform chemical produced mainly by the hydrolysis of saccharides [30].

**Figure 4:**
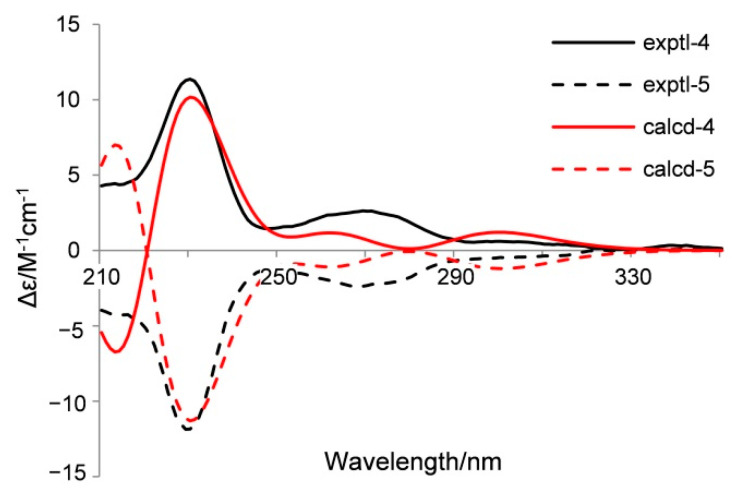
Experimental and calculated ECD spectra of **4** and **5** in MeOH.

Penoxazolones A (**4**) and B (**5**) were originally purified as a colorless oil by a series of achiral isolation. HRESIMS analysis gave the molecular formula C_18_H_16_N_2_O_4_. In accordance with the molecular formula, the ^1^H and ^13^C NMR spectra (Table 3) displayed just one set of signals assignable to one methyl, one methylene, ten methines, and six non-protonated carbons aided by DEPT and HSQC data. A detailed comparison of NMR data revealed the presence of a quinazolin-4(3*H*)-one unit [33], which was supported by the COSY correlations from H-6 thoroughly to H-9 and the HMBC correlations from H-2 to C-4 and C-10, from H-6 to C-4, C-8, and C-10, from H-8 to C-6 and C-10, and from H-9 to C-5. However, the signal for an exchangeable proton (H-3) in quinazolin-4(3H)-one was missing. The remaining NMR data corresponded to methyl 3-(4-hydroxyphenyl) propanoate [34], except for the presence of signals for a de-shielded methine group and the lack of signals for a methylene group. This methine group was adjacent to the ester carbonyl group as seen from their HMBC correlation, and it was bonded to N-3 of the quinazolin-4(3*H*)-one moiety on the basis of its HMBC correlations with C-2 and C-4. Thus, the planar structure was established, validated by other COSY and HMBC correlations (Figure 2). Before assignment of the absolute configuration, a chiral HPLC was used to detect the enantiomeric purity due to the presence of a stereogenic carbon atom (C-12). As a result, enantiomers **4** and **5** with a ratio of 1:2 were obtained (Figure S26), and they exhibited opposite optical rotations. To ascertain the absolute configurations of **4** and **5**, their ECD spectra were determined in MeOH and simulated via the time-dependent density function theory method with the same solvent. Based on the similarities between experimental and calculated ECD spectra (Figure 4), the absolute configurations of **4** and **5** were proposed as 12R and 12S, respectively. These two enantiomers might be yielded by adding quinazolin-4(3H)-one to methyl 3-(4-hydroxyphenyl)acrylate through a carbocation intermediate.

Mariculture is often threatened by harmful algal blooms and pathogenic bacteria. Compounds **1**–**5** and dankasterone A were assayed for inhibition of the three microalgae *Chattonella marina, Heterosigma akashiwo,* and *Prorocentrum donghaiense* [35], and the results are shown in Table 4. Isolates **1**, **4**, **5**, and dankasterone A could inhibit the three microalga species with the IC_50_ values ranging from 0.57 to 9.1 μg/mL. The noticeable activities of **1** and dankasterone A suggested that the steroid moiety seemed to be the key pharmacophore. In combination with the weak activities of **2**/**3**, the slightly higher activities of **1** than those of dankasterone A indicated that the 2-hydrazinylbenzoic acid unit appeared weak to increase the activities. In addition, their inhibition against four marine-derived bacterial pathogens including *Vibrio anguillarum*, *Vibrio harveyi*, *Vibrio parahaemolyticus*, and *Vibrio splendidus* was detected using the disk diffusion method [36]. We found that **1**, **4**, **5**, and dankasterone A showed moderate inhibition against *V. harveyi* and *V. parahaemolyticus*, and their inhibition zone diameters exceeded 10 mm at 20 μg/disk. The MIC values of active molecules were also measured, but only those of **4** and **5** (8 μg/mL) appeared lower than 10 μg/mL. A structure–activity relationship analysis revealed that both enantiomerization of **4** and **5** and addition of 2-hydrazinylbenzoic acid to dankasterone A had almost no influence on the antibacterial activities. In view of the above inhibitory effects of **1**, **4**, **5**, and dankasterone A on the microalgal and bacterial species, their toxicities to the marine zooplankton *Artemia salina* were also tested. All the isolates possess low toxicities, with LC_50_ values being higher that 40 μg/mL.

## 3. Materials and Methods 

### 3.1. General Experimental Procedures

Melting points were measured with an SGW X-4 micro melting point apparatus (Shanghai Precision & Scientific Instrument Co., Ltd, Shanghai, China). Optical rotations were determined on an SGW-3 polarimeter (Shanghai Shenguang Instrument Co., Ltd., Shanghai, China). UV and ECD spectra were measured on a Chirascan CD spectrometer (Applied Photophysics Ltd., Surrey, UK). IR spectra were recorded on a Nicolet iS50 FT-IR spectrometer (Thermo Fisher Scientific, Waltham, MA, USA). 1D and 2D NMR spectra were acquired on a Bruker Avance III 500 NMR spectrometer (Bruker Corp., Billerica, MA, USA). Low and high resolution ESI mass spectra were obtained on an Agilent G6230 (Agilent Technologies Inc., Santa Clara, CA, USA) or a Waters ACQUITY TOF mass spectrometer (Waters Corp., Milford, MA, USA). Agilent 1260 HPLC system (Agilent Technologies Inc., Santa Clara, CA, USA) with an Eclipse SB-C18 (5 μm, 9.4 × 250 mm) column or a (R, R) Whelk-O1 chiral column (10 μm, 4.6 × 250 mm) was used for HPLC separation. Silica gel (200–300 mesh, Qingdao Haiyang Chemical Co. Qingdao, China), RP-18 (AAG12S50, YMC Co. Ltd., Kyoto, Japan), and Sephadex LH-20 (GE Healthcare, Uppsala, Sweden) were employed for column chromatography (CC). Precoated silica gel plates (GF-254, Qingdao Haiyang Chemical Co., Qingdao, China) were used for thin-layer chromatography (TLC). Gaussian 09 software (IA32W-G09RevC.01, Gaussian, Inc., Wallingford, CT, USA) was applied to quantum chemical calculations.

### 3.2. Fungal Material and Fermentation

*Penicillium oxalicum* 13–37 was isolated from deep sea cold seep sediments. The species was identified by morphology and by analysis of the ITS regions of its rDNA, whose sequence data have been deposited at GenBank with the accession number MT898464. Its fermentation was carried out statically at room temperature for 30 days in 200 × 1 L Erlenmeyer flasks, each containing 50 g rice, 2 g glucose, 0.6 g peptone, 0.5 g yeast extract, 0.3 g monosodium glutamate, 0.1 g NaBr, 50 mL pure water, and 50 mL natural seawater from the coast of Yantai, China.

### 3.3. Extraction and Isolation

At the end of the above fermentation, the mycelia were dried in the shade and then exhaustively extracted with CH_2_Cl_2_ and MeOH (1:1, *v/v*). After removing organic solvents by evaporation under vacuum, the residue was partitioned between EtOAc and H_2_O to give an EtOAc-soluble extract (536 g). The extract was subjected to silica gel CC for separation with step-gradient solvent systems consisting of petroleum ether (PE)/EtOAc (50:1 to 0:1) and then CH_2_Cl_2_/MeOH (10:1 to 0:1). Based on TLC analysis, eight fractions (Frs. 1-8) were obtained. Fr. 5 eluted with PE/EtOAc (1:1) and was further purified by CC on RP-18 (MeOH/H_2_O, 93:7 to 49:1) to give two subfractions, Fr. 5-1 and 5-2. Fr. 5-1 eluted with MeOH/H_2_O (93:7) and was further purified by Sephadex LH-20 (CH_2_Cl_2_/MeOH, 1:1) CC and semipreparative HPLC (acetonitrile/H_2_O, 19:1 to 49:1) to obtain dankasterone A (13.4 mg). Fr. 5-2 eluted with MeOH/H_2_O (49:1) and was further purified by Sephadex LH-20 (MeOH) CC to afford **1** (9.4 mg). Fr. 6 eluted with EtOAc and was further purified by CC on RP-18 MeOH/H_2_O (7:3) and was further purified by Sephadex LH-20 (MeOH) CC as well as semipreparative HPLC (acetonitrile/H_2_O, 23:27) to obtain a mixture of **2** and **3** (totally 24.7 mg). Fr. 7 eluted with CH_2_Cl_2_/MeOH (20:1) and was further purified by CC on RP-18 (MeOH/H_2_O, 3:2) and Sephadex LH-20 (MeOH) and semipreparative HPLC (acetonitrile/H_2_O, 3:7) as well as chiral HPLC (hexane/EtOH, 7:3) to give **4** (1.2 mg) and its enantiomer **5** (1.4 mg).

Penoxahydrazone A (**1**): yellow crystals; mp 207–209 °C; [α]^27^_D_ +142 (c 0.024, MeOH); UV (MeOH) λ_max_ (Δε) 401 (4.5), 222 (4.3) nm; IR (KBr) v_max_ 3446, 2959, 1681, 1603, 1261, 1223, 1148, 1024, 978, 754 cm^−1^; ^1^H and ^13^C NMR data (Table 1); HRESIMS *m/z* 581.3357 [M + Na]^+^ (calculated for C_35_H_46_N_2_O_4_Na, 581.3355).

Penoxahydrazone B/C (**2/3**): brown powder; IR (KBr) v_max_ 3415, 2921, 2852, 1669, 1647, 1243, 1020, 755 cm^−1^; ^1^H and ^13^C NMR data (Table 2); HRESIMS m/z 283.0695 [M + Na]^+^ (calculated for C_13_H_12_N_2_O_4_Na, 283.0689) and 283.0691 [M + Na]^+^ (calculated for C_13_H_12_N_2_O_4_Na, 283.0689).

Penoxazolone A/B (**4**/**5**): colorless oil; [α]^28^_D_ +169 (*c* 0.040, MeOH) for **4** and −176 (*c* 0.050, MeOH) for **5**; UV (MeOH) λ_max_ (Δε) 226 (4.1), 272 (3.4) nm for **4** and 226 (4.3), 272 (3.6) nm for **5**; IR (KBr) *v*_max_ 3441, 2921, 2853, 1670, 1572, 1416, 1013 cm^−1^; ^1^H and ^13^C NMR data (Table 3); HRESIMS *m/z* 347.1013 [M + Na]^+^ (calculated for C_18_H_16_N_2_O_4_Na, 347.1008).

### 3.4. X-ray Crystallographic Analysis

Yellow crystals of **1** were obtained from a solution of EtOH with a drop of water. Crystallographic data were collected at 100 K using Cu Kα radiation (λ = 1.54178Å) on a Bruker APEX DUO diffractometer equipped with an APEX II CCD. The structure of **1** was solved by direct methods with the SHELXS-97 software package. All non-hydrogen atoms were refined anisotropically with SHELXL-97 and SHELXL-2014 using full-matrix least-squares, and refinements of the H atoms in calculated positions were performed using a riding model. Molecular graphics were calculated with PLATON [37]. Crystallographic data have been deposited in the Cambridge Crystallographic Data Centre as CCDC 2,024,007 for **1**. These data can be obtained free of charge via http://www.ccdc.cam.ac.uk/data_request/cif (or from the CCDC, 12 Union Road, Cambridge CB21EZ, U.K.; fax: + 44-1223-336-033; e-mail: deposit@ccdc.cam.ac.uk).

### 3.5. Crystal Data for Penoxahydrazone A (1)

Crystal data is as follows: 2(C_35_H_46_N_2_O_4_)•H_2_O, *M* = 1135.49, *a* = 14.7945(4) Å, *b* = 9.4752(2) Å, *c* = 23.3965(6) Å, *α* = 90°, *β* = 98.7230(10)°, *γ* = 90°, *V* = 3241.80(14) Å^3^, *T* = 100.(2) K, space group *P*1211, *Z* = 2, *μ*(Cu Kα) = 0.604 mm^−1^, 60,835 reflections measured, 12,787 independent reflections (*R_int_* = 0.0347). The final *R_1_* values were 0.0427 (*I* > 2*σ*(*I*)). The final *wR*(*F*^2^) values were 0.1171 (*I* > 2*σ*(*I*)). The final *R_1_* values were 0.0433 (all data). The final *wR*(*F*^2^) values were 0.1179 (all data). The goodness of fit on *F*^2^ was 1.032. Flack parameter = 0.01(4).

### 3.6. Computational Details

Conformational searches for compounds **2**–**5** were operated via the Dreiding force field in MarvinSketch software (optimization limit = normal, diversity limit = 0.1; MarvinSketch with Calculator Plugins for Structure Property Prediction and Calculation, Marvin, Version 6.2.2, 2014, ChemAxon, Budapest, Hungary). Regardless of the rotation of methyl and hydroxy groups, the energy-minimized conformers (Figures S1–S4) within a 3 kcal/mol energy threshold from the global minimum were generated after conformational optimization at the B3LYP/6-31+G(d,p) level in DMSO for **2** and **3** and at the B3LYP/6-31G(d) level in MeOH for **4** and **5** using Gaussian 09 software [31]. The ^1^H and ^13^C NMR data of each conformer of **2** and **3** were computed at the B3LYP/6-31+G(d,p) level in DMSO through the gauge-independent atomic orbital (GIAO) method, while the ECD spectrum of each conformer of **4** and **5** was calculated at the B3LYP/6-31G(d) level in MeOH via the time-dependent density function theory (TD-DFT) method and then drawn by SpecDis software with sigma = 0.25 and UV-shift = −8 nm. The overall calculated ^1^H and ^13^C NMR data and ECD curves of each compound were produced by Boltzmann weighting. All of the above calculations were performed with the integral equation formalism variant (IEF) of the polarizable continuum model (PCM) as implemented in Gaussian 09.

## 4. Conclusions

Five new dinitrogen-bearing metabolites have been isolated and identified from a cold-seep-derived strain of *P. oxalicum*, and they display high novelty due to the unprecedented linkages. As phenylhydrazone derivatives, **1** features a special steroid framework, while tautomers **2** and **3** contain a furan ring bonding to the azo group. It seems that this fungal strain has the ability to form phenylhydrazones through adding 2-hydrazinylbenzoic acid to ketones or aldehydes. The discovery of penoxahydrazones A–C (**1**–**3**) adds greatly to the diversity of phenylhydrazone derivatives. As quinazoline derivatives, enantiomers **4** and **5** possess a unique linkage between quinazoline alkaloid and cinnamic acid units. These metabolites with more or less antimicroalgal and antibacterial activities are structurally different from those isolated from the *P. oxalicum* strains of other origin, which demonstrates that deep-sea-derived fungi actually feature the potential to produce novel metabolites and further underpins the significance of chemically exploring the extreme-environment-derived fungi, such as those from the deep sea cold seep.

## Figures and Tables

**Table 1:** ^1^H and ^13^C NMR data for compound **1** (in CDCl_3_, 500 MHz).

Pos	*δ*_c_, Type	*δ*_H_ (*J* in Hz)
1a	37.6, CH_2_	1.98, m
1b		1.90, m
2a	20.9, CH_2_	2.60, m
2b		2.40, m
3	144.4, C	
4	131.7, CH	7.08, s
5	141.6, C	
6	199.2, C	
7a	41.3, CH_2_	2.61, d (16.1)
7b		2.53, d (16.1)
8	62.8, C	
9	49.5, CH	2.85, t (9.4)
10	35.6, C	
11a	25.6, CH_2_	1.92, m
11b		1.81, m
12a	39.2, CH_2_	1.75, m
12b		1.62, dt (12.5, 8.1)
13	53.8, C	
14	215.9, C	
15a	38.2, CH_2_	2.56, m
15b		2.46, m
16a	23.2, CH_2_	1.86, m
16b		1.74, m
17	50.8, CH	1.39, br d (12.7)
18	17.3, CH_3_	1.00, s
19	24.5, CH_3_	1.16, s
20	37.5, CH	2.39, m
21	23.6, CH_3_	1.07, d (7.0)
22	132.8, CH	5.25, dd (15.5, 4.6)
23	135.0, CH	5.28, dd (15.5, 5.0)
24	43.4, CH	1.87, m
25	33.2, CH	1.47, octet (6.8)
26	19.8, CH_3_	0.82, d (6.8)
27	20.2, CH_3_	0.84, d (6.8)
28	17.8, CH_3_	0.92, d (6.8)
30		11.07, s
31	147.3, C	
32	114.1, CH	7.66, d (8.5)
33	135.8, CH	7.45, dd (8.5, 7.1)
34	119.1, CH	6.84, dd (7.8, 7.1)
35	131.7, CH	7.98, d (7.8)
36	109.4, C	
37	172.4, C	

**Table 2:** ^1^H and ^13^C NMR data for compounds **2** and **3** (in DMSO-*d*_6_).

Pos	2		3	
	*δ*_c_, Type	*δ*_H_ (*J* in Hz)	*δ*_c_, Type	*δ*_H_ (*J* in Hz)
1	169.6, C		169.5, C	
2	111.0, C		110.1, C	
3	131.3, CH	7.89, dd (7.9, 1.3)	131.2, CH	7.84, dd (7.9, 1.4)
4	118.3, CH	6.84, br dd (7.9, 7.1)	117.6, CH	6.79, dd (7.9, 7.1)
5	134.4, CH	7.48, br dd (8.4, 7.1)	134.4, CH	7.48, br dd (8.4, 7.1)
6	112.9, CH	7.68, br d (8.4)	113.1, CH	7.59, br d (8.4)
7	147.1, C		146.9, C	
8		12.33, br s		11.23, br s
10	125.4, CH	7.27, s	131.4, CH	8.03, s
11	146.8, C		149.4, C	
12	113.7, CH	7.01, d (3.3)	112.0, CH	6.65, d (3.3)
13	108.9, CH	6.54, d (3.3)	109.2, CH	6.40, d (3.3)
14	157.2, C		156.7, C	
15	56.0, CH_2_	4.57, s	55.8, CH_2_	4.44, s

**Table 3:** ^1^H and ^13^C NMR data for compounds **4** and **5** (in DMSO-*d*_6_).

Pos	*δ*_c_, Type	*δ*_H_ (*J* in Hz)
2	147.2, CH	8.01, s
4	159.9, C	
5	121.1, C	
6	126.1, CH	8.12, dd (7.9, 1.2)
7	127.4, CH	7.55, dd (7.9, 7.1)
8	134.8, CH	7.83, ddd (8.1, 7.1, 1.2)
9	127.2, CH	7.62, d (8.1)
10	147.4, C	
11	169.3, C	
12	60.4, CH	5.41, dd (11.1, 4.9)
13a	33.3, CH_2_	3.41, dd (14.3, 4.9)
13b		3.32, dd (14.3, 11.1)
14	126.1, C	
15	129.8, CH	6.87, d (8.4)
16	115.3, CH	6.54, d (8.4)
17	156.2, C	
18	115.3, CH	6.54, d (8.4)
19	129.8, CH	6.87, d (8.4)
20	52.6, CH_3_	3.69, s

**Table 4:** Inhibition of marine microalgae and marine-derived bacteria.

Compounds	IC_50_ (μg/mL)			Inhibitory Zone Diameter (mm) at 20 μg/disk				LC_50_ (μg/mL)
	*C. marina*	*H. akashiwo*	*P. donghaiense*	*V. anguillarum*	*V. harveyi*	*V. parahaemolyticus*	*V. splendidus*	*A. salina*
1	1.2	3.7	0.68	0	11	12	0	58
2/3	17	>100	5.4	0	0	7.0	9.7	>100
4	2.8	8.1	0.57	7.0	13	11	7.3	>100
5	9.1	9.0	1.2	0	13	11	7.0	>100
dankasterone A	1.9	4.6	1.0	7.0	12	12	0	43
K_2_Cr_2_O_7_	0.60	2.4	1.2					18
chloramphenicol				18	29	28	23	

## Data Availability

The data presented in this study are available in the supplementary material.

## Supplementary Materials

The following are available online at https://www.mdpi.com/1660-3397/19/1/9/s1, Tables S1–S4: Sarotti′s DP4+ sheets. Figures S1–S4: energy-minimized conformers, Figures S5–S26: 1D/2D NMR and HRMS spectra.
