# Supplementary material for: Phenylhydrazone and Quinazoline Derivatives from the Cold-Seep-Derived Fungus Penicillium oxalicum
Source: Mar Drugs. 2020 Dec 28;19(1):9. doi: 10.3390/md19010009 (PMC7824341; doi:10.3390/md19010009)

# checkCIF/PLATON report

Structure factors have been supplied for datablock(s) yt_jny1

THIS REPORT IS FOR GUIDANCE ONLY. IF USED AS PART OF A REVIEW PROCEDURE FOR PUBLICATION, IT SHOULD NOT REPLACE THE EXPERTISE OF AN EXPERIENCED CRYSTALLOGRAPHIC REFEREE.

No syntax errors found. CIF dictionary Interpreting this report

## Datablock: yt_jny1

---

```
Bond precision:  C-C = 0.0038 A         Wavelength=1.54178

Cell:            a=14.7945(4)     b=9.4752(2)        c=23.3965(6)
                 alpha=90         beta=98.723(1)     gamma=90
Temperature:     100 K

                Calculated                  Reported
Volume          3241.81(14)                 3241.80(14)
Space group     P 21                        P 1 21 1
Hall group      P 2yb                       P 2yb
Moiety formula  2(C35 H46 N2 O4), O         2(C35 H46 N2 O4), H2 O
Sum formula     C70 H92 N4 O9               C70 H94 N4 O9
Mr              1133.48                     1135.49
Dx,g cm-3       1.161                       1.163
Z               2                           2
Mu (mm-1)       0.603                       0.604
F000            1224.0                      1228.0
F000'           1227.49
h,k,lmax        18,11,28                    18,11,28
Nref            12834[ 6827]                12787
Tmin,Tmax       0.700,0.924                 0.700,0.930
Tmin'           0.624

Correction method= # Reported T Limits: Tmin=0.700 Tmax=0.930
AbsCorr = MULTI-SCAN

Data completeness= 1.87/1.00      Theta(max)= 72.330

R(reflections)= 0.0427( 12556)    wR2(reflections)= 0.1179( 12787)

S = 1.032                 Npar= 773
```

---

The following ALERTS were generated. Each ALERT has the format
**test-name_ALERT_alert-type_alert-level**.
Click on the hyperlinks for more details of the test.

## Alert level B

```
PLAT097_ALERT_2_B Large Reported Max.  (Positive) Residual Density       0.98 eA-3
PLAT420_ALERT_2_B D-H Without Acceptor       O8        --H91      .     Please Check
PLAT430_ALERT_2_B Short Inter D...A Contact  O7        ..O9A      .       2.82 Ang.
                                             1-x,-1/2+y,1-z  =      2_646 Check
PLAT430_ALERT_2_B Short Inter D...A Contact  O7        ..O9       .       2.84 Ang.
                                                    x,y,1+z  =      1_556 Check
```

## Alert level C

```
DIFMX02_ALERT_1_C  The maximum difference density is > 0.1*ZMAX*0.75
            The relevant atom site should be identified.
PLAT041_ALERT_1_C Calc. and Reported SumFormula    Strings  Differ     Please Check
PLAT043_ALERT_1_C Calculated and Reported Mol. Weight Differ by ..       2.01 Check
PLAT094_ALERT_2_C Ratio of Maximum / Minimum Residual Density ....       3.05 Report
PLAT911_ALERT_3_C Missing FCF Refl Between Thmin & STh/L=    0.600         10 Report
PLAT913_ALERT_3_C Missing # of Very Strong Reflections in FCF ....          9 Note
PLAT918_ALERT_3_C Reflection(s) with I(obs) much Smaller I(calc) .          1 Check
PLAT934_ALERT_3_C Number of (Iobs-Icalc)/Sigma(W) > 10 Outliers ..          1 Check
PLAT975_ALERT_2_C Check Calcd Resid. Dens.  1.02A   From O9              1.02 eA-3
```

## Alert level G

```
FORMU01_ALERT_2_G  There is a discrepancy between the atom counts in the
            _chemical_formula_sum and the formula from the _atom_site* data.
            Atom count from _chemical_formula_sum:C70 H94 N4 O9
            Atom count from the _atom_site data:  C70 H92 N4 O9
CELLZ01_ALERT_1_G Difference between formula and atom_site contents detected.
CELLZ01_ALERT_1_G WARNING: H atoms missing from atom site list. Is this intentional?
           From the CIF: _cell_formula_units_Z    2
           From the CIF: _chemical_formula_sum  C70 H94 N4 O9
           TEST: Compare cell contents of formula and atom_site data

           atom    Z*formula  cif sites diff
           C        140.00    140.00    0.00
           H        188.00    184.00    4.00
           N          8.00      8.00    0.00
           O         18.00     18.00    0.00
PLAT007_ALERT_5_G Number of Unrefined Donor-H Atoms ..............          3 Report
PLAT042_ALERT_1_G Calc. and Reported MoietyFormula Strings  Differ     Please Check
PLAT063_ALERT_4_G Crystal Size Possibly too Large for Beam Size ..       0.70 mm
PLAT066_ALERT_1_G Predicted and Reported Tmin&Tmax Range Identical          ? Check
PLAT068_ALERT_1_G Reported F000 Differs from Calcd (or Missing)...     Please Check
PLAT302_ALERT_4_G Anion/Solvent/Minor-Residue Disorder (Resd  3  )       100% Note
PLAT302_ALERT_4_G Anion/Solvent/Minor-Residue Disorder (Resd  4  )       100% Note
PLAT304_ALERT_4_G Non-Integer Number of Atoms in ..... (Resd  3  )       0.82 Check
PLAT304_ALERT_4_G Non-Integer Number of Atoms in ..... (Resd  4  )       0.18 Check
PLAT311_ALERT_2_G Isolated Disordered Oxygen Atom (No H's ?) .....         O9 Check
PLAT311_ALERT_2_G Isolated Disordered Oxygen Atom (No H's ?) .....        O9A Check
PLAT790_ALERT_4_G Centre of Gravity not Within Unit Cell: Resd.  #          3 Note
              O
PLAT791_ALERT_4_G Model has Chirality at C3          (Sohnke SpGr)          R Verify
PLAT791_ALERT_4_G Model has Chirality at C6          (Sohnke SpGr)          R Verify
PLAT791_ALERT_4_G Model has Chirality at C7          (Sohnke SpGr)          R Verify
PLAT791_ALERT_4_G Model has Chirality at C8          (Sohnke SpGr)          R Verify
PLAT791_ALERT_4_G Model has Chirality at C9          (Sohnke SpGr)          R Verify
PLAT791_ALERT_4_G Model has Chirality at C10         (Sohnke SpGr)          R Verify
PLAT791_ALERT_4_G Model has Chirality at C11         (Sohnke SpGr)          R Verify
PLAT791_ALERT_4_G Model has Chirality at C38         (Sohnke SpGr)          R Verify
PLAT791_ALERT_4_G Model has Chirality at C41         (Sohnke SpGr)          R Verify
PLAT791_ALERT_4_G Model has Chirality at C42         (Sohnke SpGr)          R Verify
```

```
PLAT791_ALERT_4_G Model has Chirality at C43         (Sohnke SpGr)          R Verify
PLAT791_ALERT_4_G Model has Chirality at C44         (Sohnke SpGr)          R Verify
PLAT791_ALERT_4_G Model has Chirality at C45         (Sohnke SpGr)          S Verify
PLAT791_ALERT_4_G Model has Chirality at C70         (Sohnke SpGr)          R Verify
PLAT883_ALERT_1_G No Info/Value for _atom_sites_solution_primary .     Please Do !
PLAT910_ALERT_3_G Missing # of FCF Reflection(s) Below Theta(Min).          2 Note
PLAT912_ALERT_4_G Missing # of FCF Reflections Above STh/L=  0.600          3 Note
PLAT978_ALERT_2_G Number C-C Bonds with Positive Residual Density.         12 Info
PLAT992_ALERT_5_G Repd & Actual _reflns_number_gt Values Differ by          2 Check
```

---

```
   0 ALERT level A = Most likely a serious problem - resolve or explain
   4 ALERT level B = A potentially serious problem, consider carefully
   9 ALERT level C = Check. Ensure it is not caused by an omission or oversight
  34 ALERT level G = General information/check it is not something unexpected

   9 ALERT type 1 CIF construction/syntax error, inconsistent or missing data
  10 ALERT type 2 Indicator that the structure model may be wrong or deficient
   5 ALERT type 3 Indicator that the structure quality may be low
  21 ALERT type 4 Improvement, methodology, query or suggestion
   2 ALERT type 5 Informative message, check
```

---

### Validation response form

Please find below a validation response form (VRF) that can be filled in and pasted into your CIF.

```
# start Validation Reply Form
_vrf_DIFMX02_yt_jny1
;
PROBLEM: The maximum difference density is > 0.1*ZMAX*0.75
RESPONSE: ...
;
_vrf_PLAT041_yt_jny1
;
PROBLEM: Calc. and Reported SumFormula    Strings  Differ     Please Check
RESPONSE: ...
;
_vrf_PLAT043_yt_jny1
;
PROBLEM: Calculated and Reported Mol. Weight Differ by ..       2.01 Check
RESPONSE: ...
;
_vrf_PLAT094_yt_jny1
;
PROBLEM: Ratio of Maximum / Minimum Residual Density ....       3.05 Report
RESPONSE: ...
;
_vrf_PLAT911_yt_jny1
;
PROBLEM: Missing FCF Refl Between Thmin & STh/L=    0.600         10 Report
RESPONSE: ...
;
_vrf_PLAT913_yt_jny1
;
PROBLEM: Missing # of Very Strong Reflections in FCF ....          9 Note
RESPONSE: ...
;
_vrf_PLAT918_yt_jny1
;
PROBLEM: Reflection(s) with I(obs) much Smaller I(calc) .          1 Check
```

```
RESPONSE: ...
;
_vrf_PLAT934_yt_jny1
;
PROBLEM: Number of (Iobs-Icalc)/Sigma(W) > 10 Outliers ..          1 Check
RESPONSE: ...
;
_vrf_PLAT975_yt_jny1
;
PROBLEM: Check Calcd Resid. Dens.  1.02A   From O9              1.02 eA-3
RESPONSE: ...
;
# end Validation Reply Form
```

---

It is advisable to attempt to resolve as many as possible of the alerts in all categories. Often the minor alerts point to easily fixed oversights, errors and omissions in your CIF or refinement strategy, so attention to these fine details can be worthwhile. In order to resolve some of the more serious problems it may be necessary to carry out additional measurements or structure refinements. However, the purpose of your study may justify the reported deviations and the more serious of these should normally be commented upon in the discussion or experimental section of a paper or in the "special_details" fields of the CIF. checkCIF was carefully designed to identify outliers and unusual parameters, but every test has its limitations and alerts that are not important in a particular case may appear. Conversely, the absence of alerts does not guarantee there are no aspects of the results needing attention. It is up to the individual to critically assess their own results and, if necessary, seek expert advice.

**Publication of your CIF in IUCr journals**

A basic structural check has been run on your CIF. These basic checks will be run on all CIFs submitted for publication in IUCr journals (*Acta Crystallographica*, *Journal of Applied Crystallography*, *Journal of Synchrotron Radiation*); however, if you intend to submit to *Acta Crystallographica Section C* or *E* or *IUCrData*, you should make sure that full publication checks are run on the final version of your CIF prior to submission.

**Publication of your CIF in other journals**

Please refer to the *Notes for Authors* of the relevant journal for any special instructions relating to CIF submission.

---

**PLATON version of 10/08/2020; check.def file version of 06/08/2020**

**Datablock yt_jny1** - ellipsoid plot

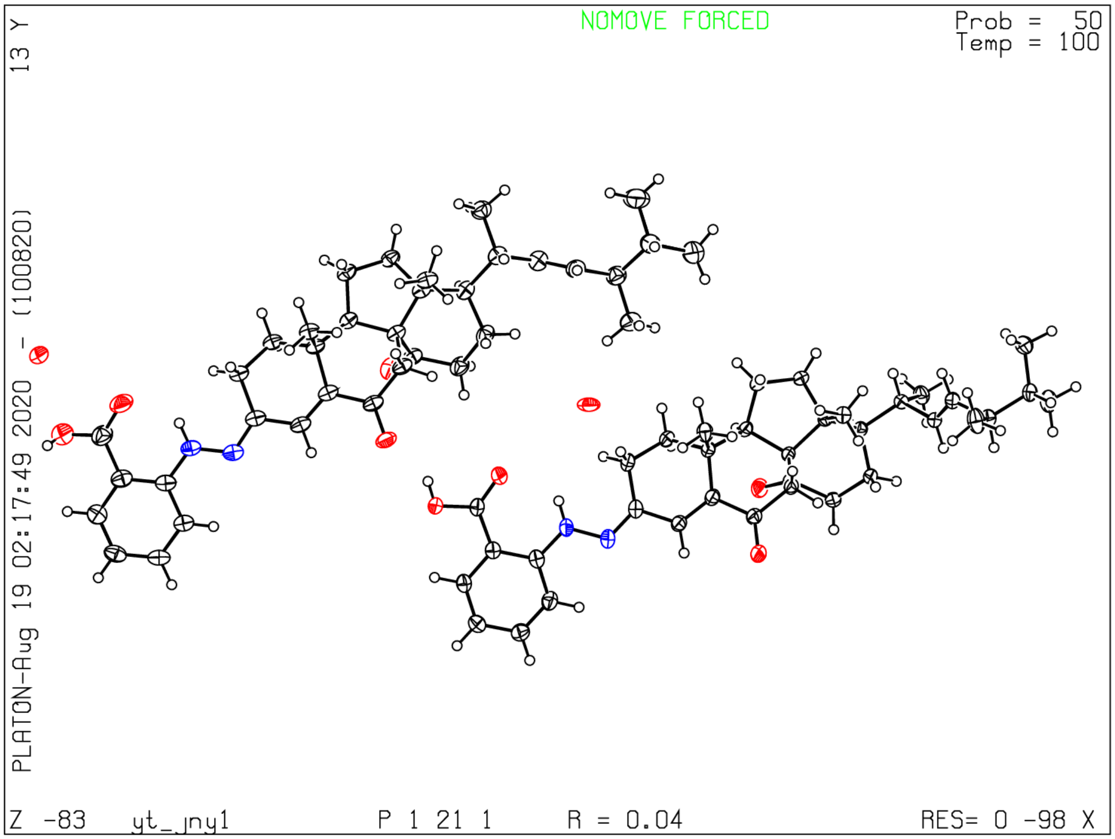

Supplement: Supplementary file 1 [file marinedrugs-19-00009-s001.zip › R1-marinedrugs-1044771-supplementary/checkcif-compound 1.pdf]
