# Supplementary material for: Phenylhydrazone and Quinazoline Derivatives from the Cold-Seep-Derived Fungus Penicillium oxalicum
Source: Mar Drugs. 2020 Dec 28;19(1):9. doi: 10.3390/md19010009 (PMC7824341; doi:10.3390/md19010009)
Supplement: Supplementary file 1 [file marinedrugs-19-00009-s001.zip › R1-marinedrugs-1044771-supplementary/R1-SI.docx]

**Supporting Information**

Phenylhydrazone and Quinazoline Derivatives from the Cold-Seep-Derived Fungus *Penicillium oxalicum*

Ya-Ping Liu ^1,2^, Sheng-Tao Fang ^1^, Zhen-Zhen Shi ^1^, Bin-Gui Wang ^3^, Xiao-Nian Li ^4^ and Nai-Yun Ji ^1,^*

^1^ Yantai Institute of Coastal Zone Research, Center for Ocean Mega-Science, Chinese Academy of Sciences, Yantai 264003, China; ypliu@yic.ac.cn (Y.-P.L.); stfang@yic.ac.cn (S.-T.F.); zzshi@yic.ac.cn (Z.-Z.S.)

^2^ University of Chinese Academy of Sciences, Beijing 100049, China

^3^ Laboratory of Marine Biology and Biotechnology of the Qingdao National Laboratory for Marine Science and Technology, Key Laboratory of Experimental Marine Biology at the Institute of Oceanology, Center for Ocean Mega-Science, Chinese Academy of Sciences, Qingdao 266071, China; wangbg@ms.qdio.ac.cn (B.-G.W.)

^4^ Kunming Institute of Botany, Chinese Academy of Sciences, Kunming 650201, China; lixiaonian@mail.kib.ac.cn (X.-N.L.)

***** Correspondence: nyji@yic.ac.cn; Tel.: +86-535-210-9176

**Contents**

**Table S1.** Sarotti′s DP4+ sheet for ^1^H NMR data of compound **2**.

**Table S2.** Sarotti′s DP4+ sheet for ^1^H NMR data of compound **3**.

**Table S3.** Sarotti′s DP4+ sheet for ^13^C NMR data of compound **2**.

**Table S4.** Sarotti′s DP4+ sheet for ^13^C NMR data of compound **3**.

**Figure S1.** Energy-minimized conformers (**2a**–**2h** with Boltzmann populations) of compound **2** within a 3 kcal/mol energy threshold from the global minimum optimized at the B3LYP/6-31+G(d,p) level in DMSO.

**Figure S2.** Energy-minimized conformers (**3a**–**3h** with Boltzmann populations) of compound **3** within a 3 kcal/mol energy threshold from the global minimum optimized at the B3LYP/6-31+G(d,p) level in DMSO.

**Figure S3.** Energy-minimized conformers (**4a**–**4t** with Boltzmann populations) of compound **4** within a 3 kcal/mol energy threshold from the global minimum optimized at the B3LYP/6-31G(d) level in MeOH.

**Figure S4.** Energy-minimized conformers (**5a**–**5t** with Boltzmann populations) of compound **5** within a 3 kcal/mol energy threshold from the global minimum optimized at the B3LYP/6-31G(d) level in MeOH.

**Figure S5.** ^1^H NMR spectrum of compound **1** in CDCl_3_.

**Figure S6.** ^13^C NMR and DEPT spectra of compound **1** in CDCl_3_.

**Figure S7.** HSQC spectrum of compound **1** in CDCl_3_.

**Figure S8.** HMBC spectrum of compound **1** in CDCl_3_.

**Figure S9.** COSY spectrum of compound **1** in CDCl_3_.

**Figure S10.** NOESY spectrum of compound **1** in CDCl_3_.

**Figure S11.** HRESIMS spectrum of compound **1**.

**Figure S12.** ^1^H NMR spectrum of compounds **2** and **3** in DMSO-*d*_6_.

**Figure S13.** ^13^C NMR and DEPT spectra of compounds **2** and **3** in DMSO-*d*_6_.

**Figure S14.** HSQC spectrum of compounds **2** and **3** in DMSO-*d*_6_.

**Figure S15.** HMBC spectrum of compounds **2** and **3** in DMSO-*d*_6_.

**Figure S16.** COSY spectrum of compounds **2** and **3** in DMSO-*d*_6_.

**Figure S17.** NOESY spectrum of compounds **2** and **3** in DMSO-*d*_6_.

**Figure S18.** LC-HRESIMS spectrum of compounds **2** and **3**.

**Figure S19.** ^1^H NMR spectrum of compound compounds **4** and **5** in DMSO-*d*_6_.

**Figure S20.** ^13^C NMR and DEPT spectra of compounds **4** and **5** in DMSO-*d*_6_.

**Figure S21.** HSQC spectrum of compounds **4** and **5** in DMSO-*d*_6_.

**Figure S22.** HMBC spectrum of compounds **4** and **5** in DMSO-*d*_6_.

**Figure S23.** COSY spectrum of compounds **4** and **5** in DMSO-*d*_6_.

**Figure S24.** NOESY spectrum of compounds **4** and **5** in DMSO-*d*_6_.

**Figure S25.** HRESIMS spectrum of compounds **4** and **5**.

**Figure S26.** Chiral HPLC spectrum of compounds **4** and **5**.

**Table S1.** Sarotti′s DP4+ sheet for ^1^H NMR data of compound **2**.


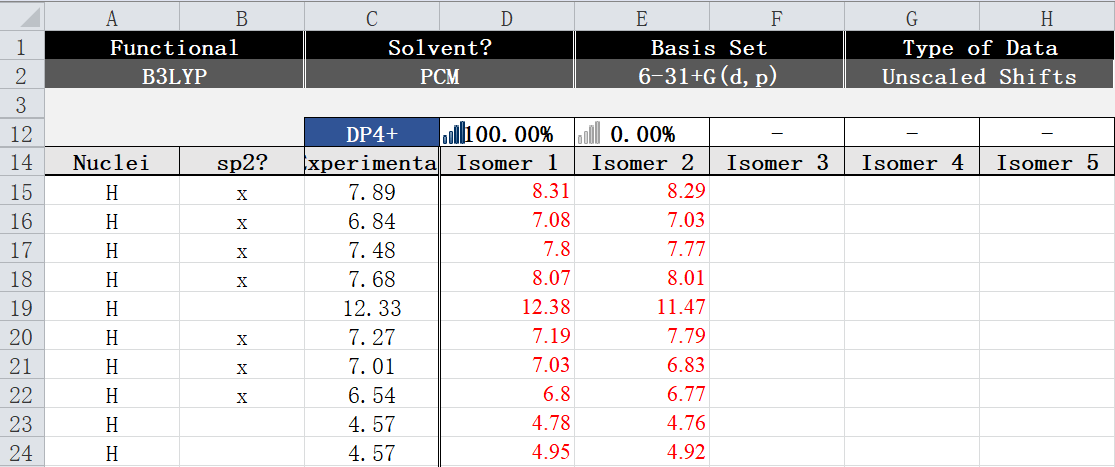


**Table S2.** Sarotti′s DP4+ sheet for ^1^H NMR data of compound **3**.


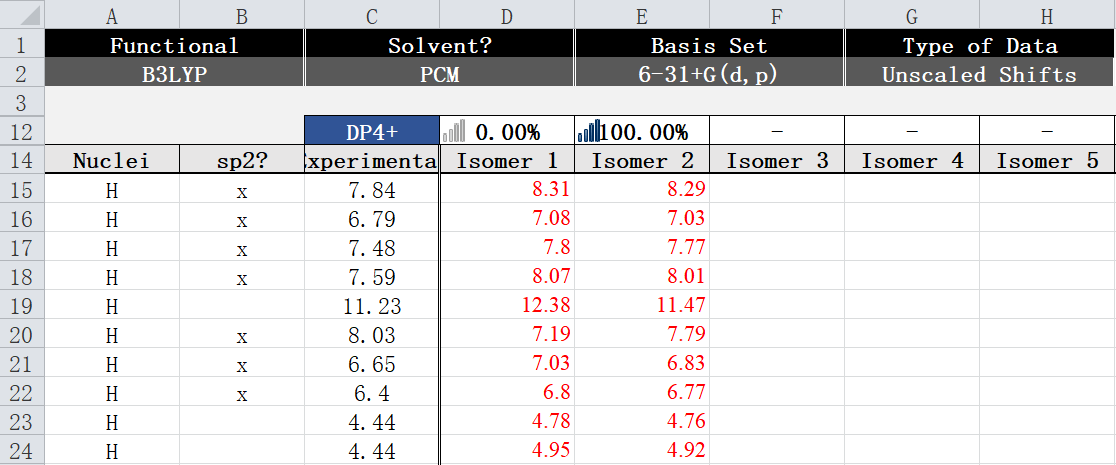


**Table S3.** Sarotti′s DP4+ sheet for ^13^C NMR data of compound **2**.


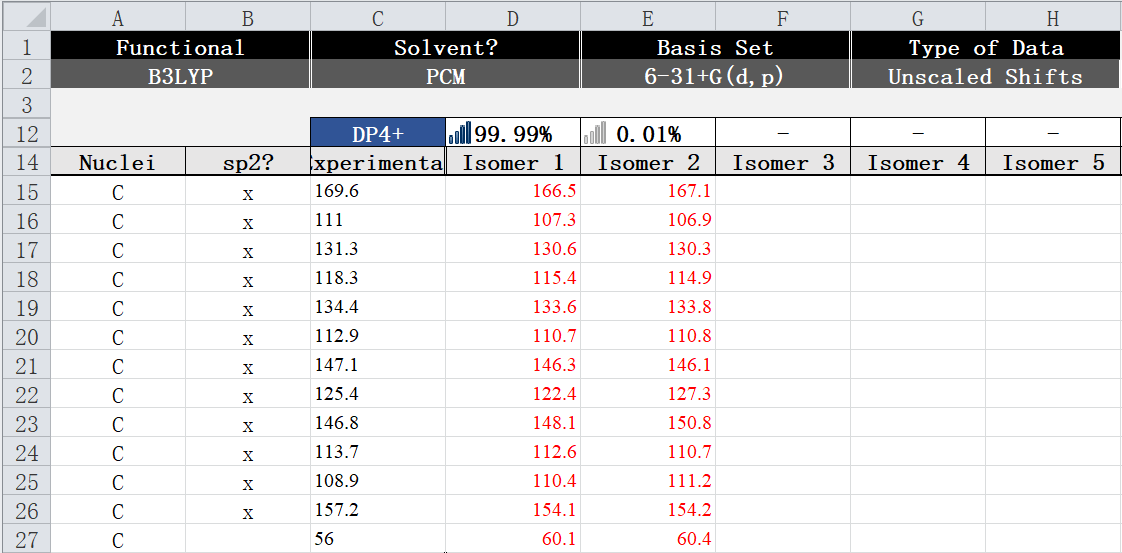


**Table S4.** Sarotti′s DP4+ sheet for ^13^C NMR data of compound **3**.


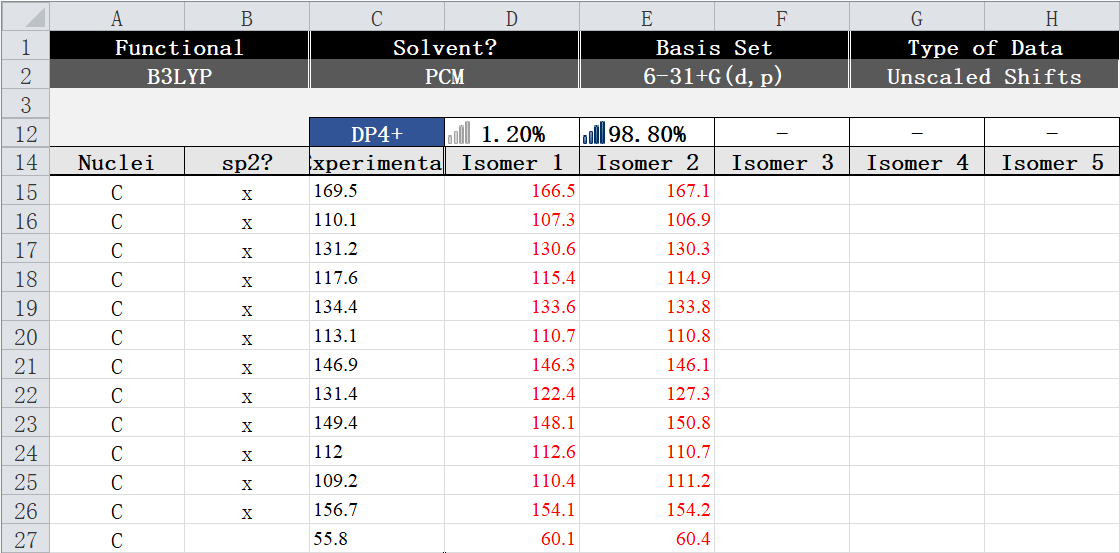


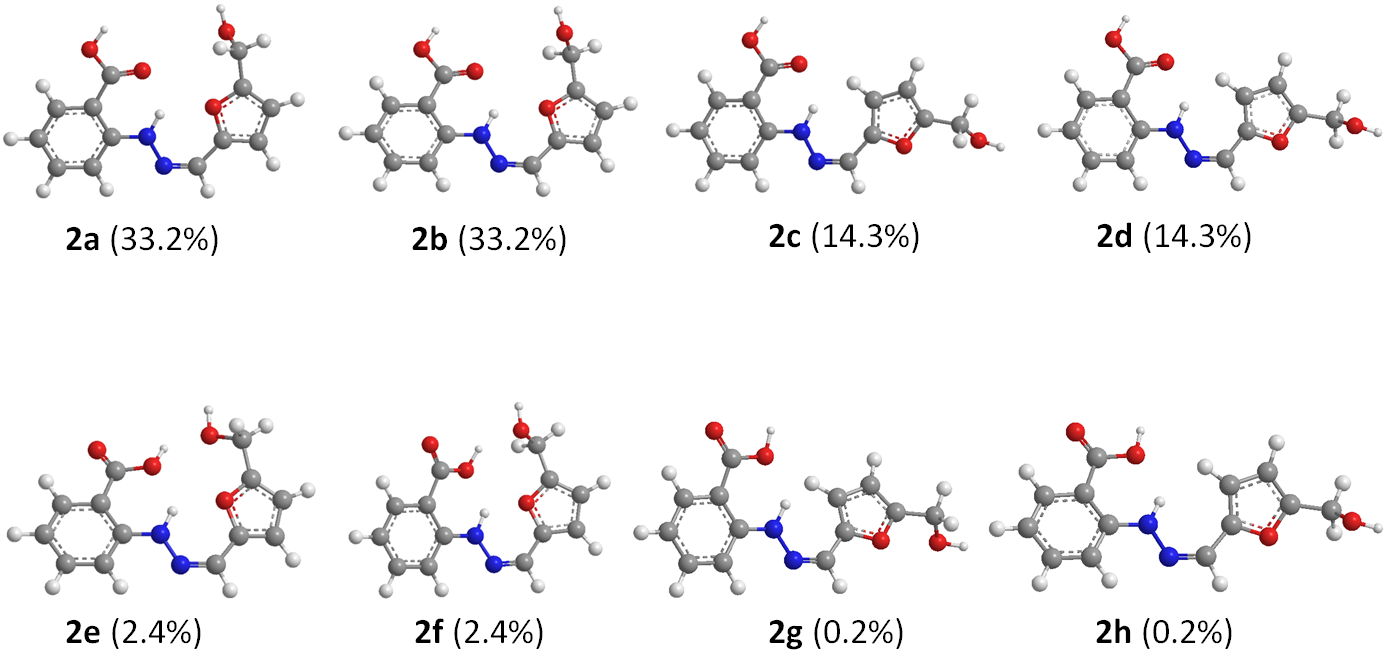


**Figure S1.** Energy-minimized conformers (**2a**–**2h** with Boltzmann populations) of compound **2** within a 3 kcal/mol energy threshold from the global minimum optimized at the B3LYP/6-31+G(d,p) level in DMSO.


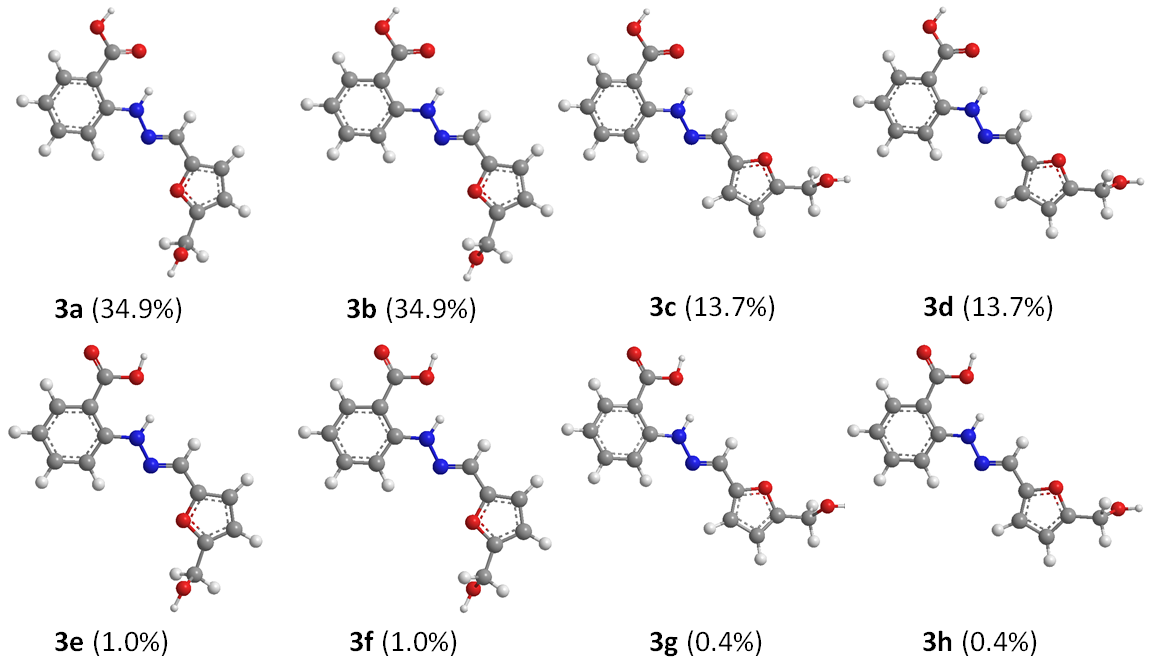


**Figure S2.** Energy-minimized conformers (**3a**–**3h** with Boltzmann populations) of compound **3** within a 3 kcal/mol energy threshold from the global minimum optimized at the B3LYP/6-31+G(d,p) level in DMSO.


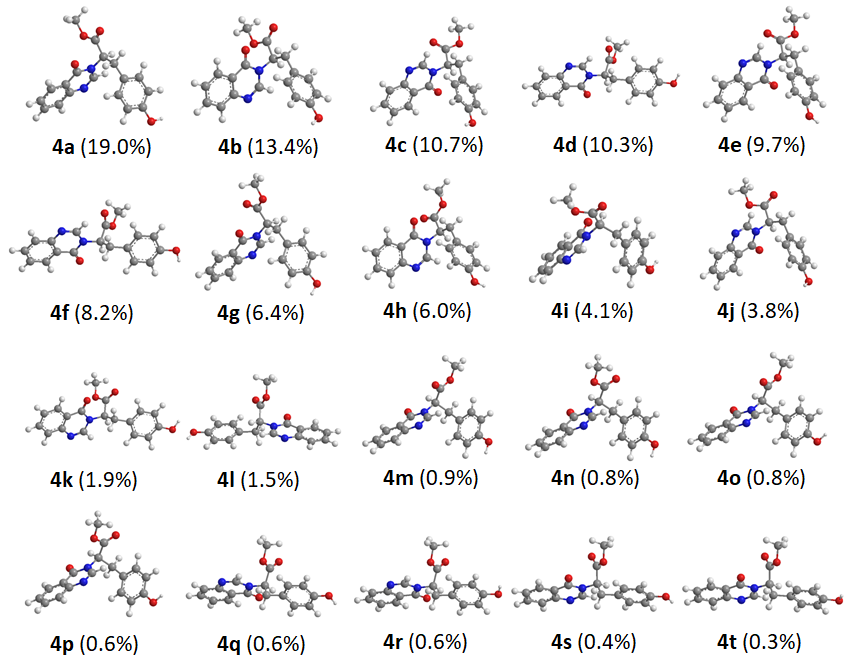


**Figure S3.** Energy-minimized conformers (**4a**–**4t** with Boltzmann populations) of compound **4** within a 3 kcal/mol energy threshold from the global minimum optimized at the B3LYP/6-31G(d) level in MeOH (note: population differences from those of **5** are just a problem of approximations).


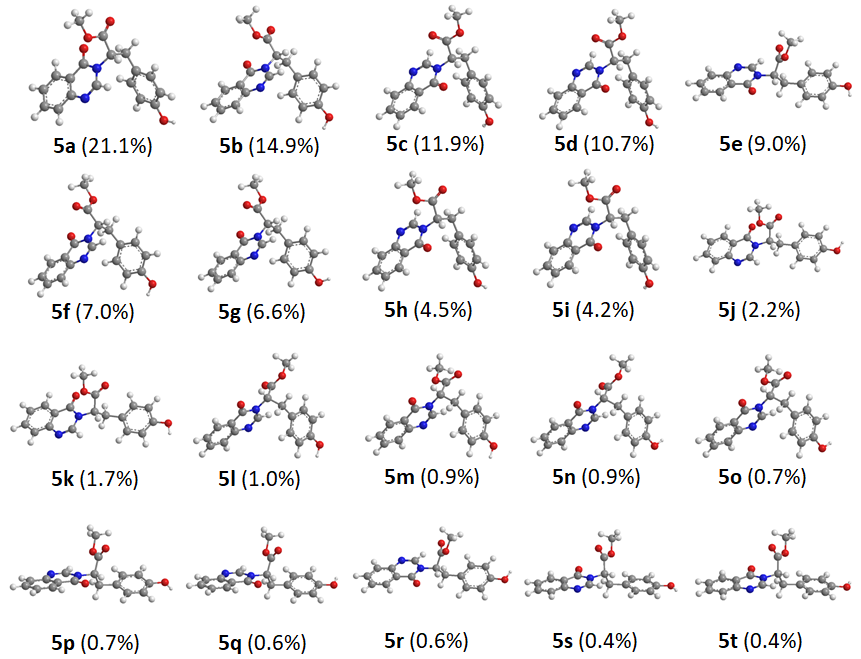


**Figure S4.** Energy-minimized conformers (**5a**–**5t** with Boltzmann populations) of compound **5** within a 3 kcal/mol energy threshold from the global minimum optimized at the B3LYP/6-31G(d) level in MeOH (note: population differences from those of **4** are just a problem of approximations).


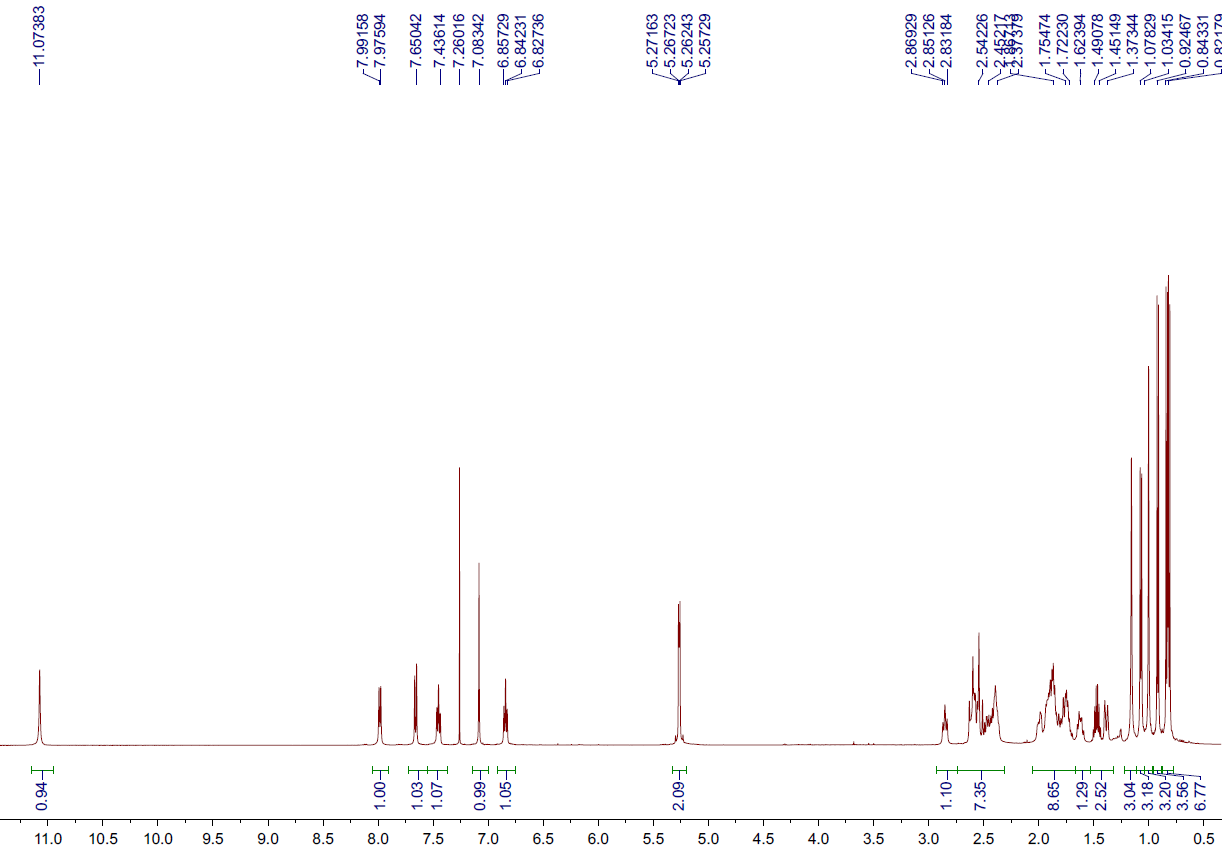


**Figure S5.** ^1^H NMR spectrum of compound **1** in CDCl_3_.


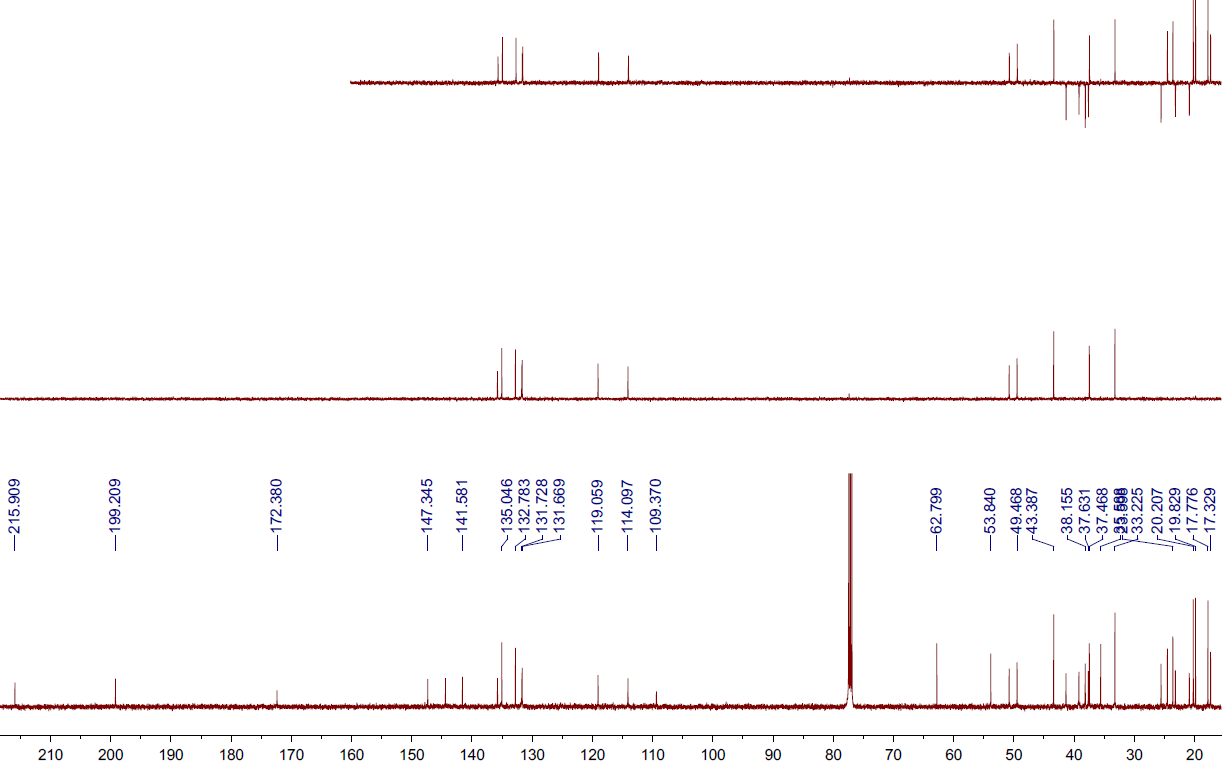


**Figure S6.** ^13^C NMR and DEPT spectra of compound **1** in CDCl_3_.


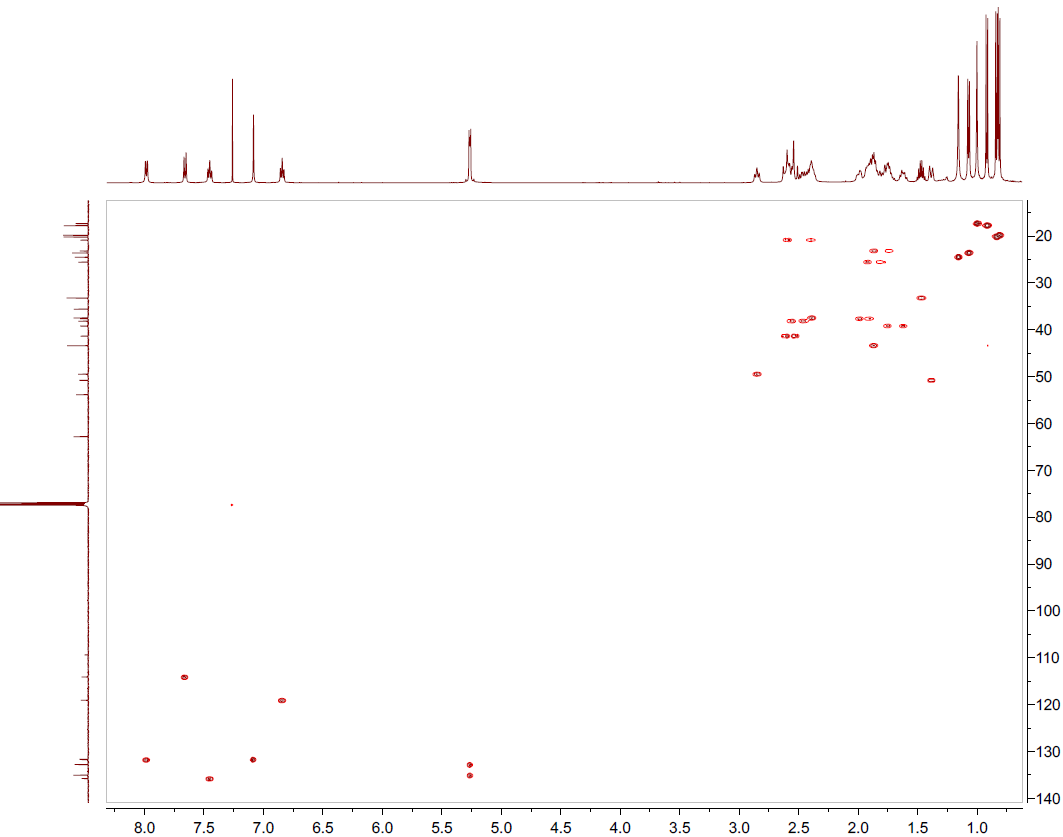


**Figure S7.** HSQC spectrum of compound **1** in CDCl_3_.


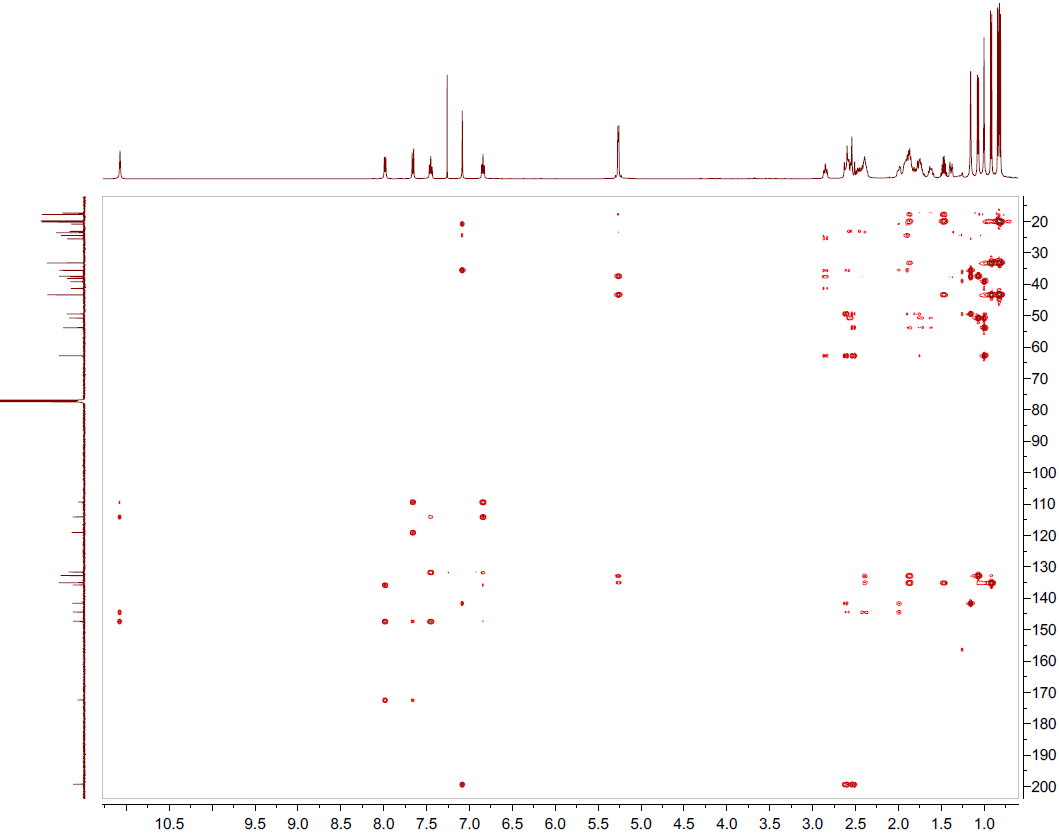


**Figure S8.** HMBC spectrum of compound **1** in CDCl_3_.


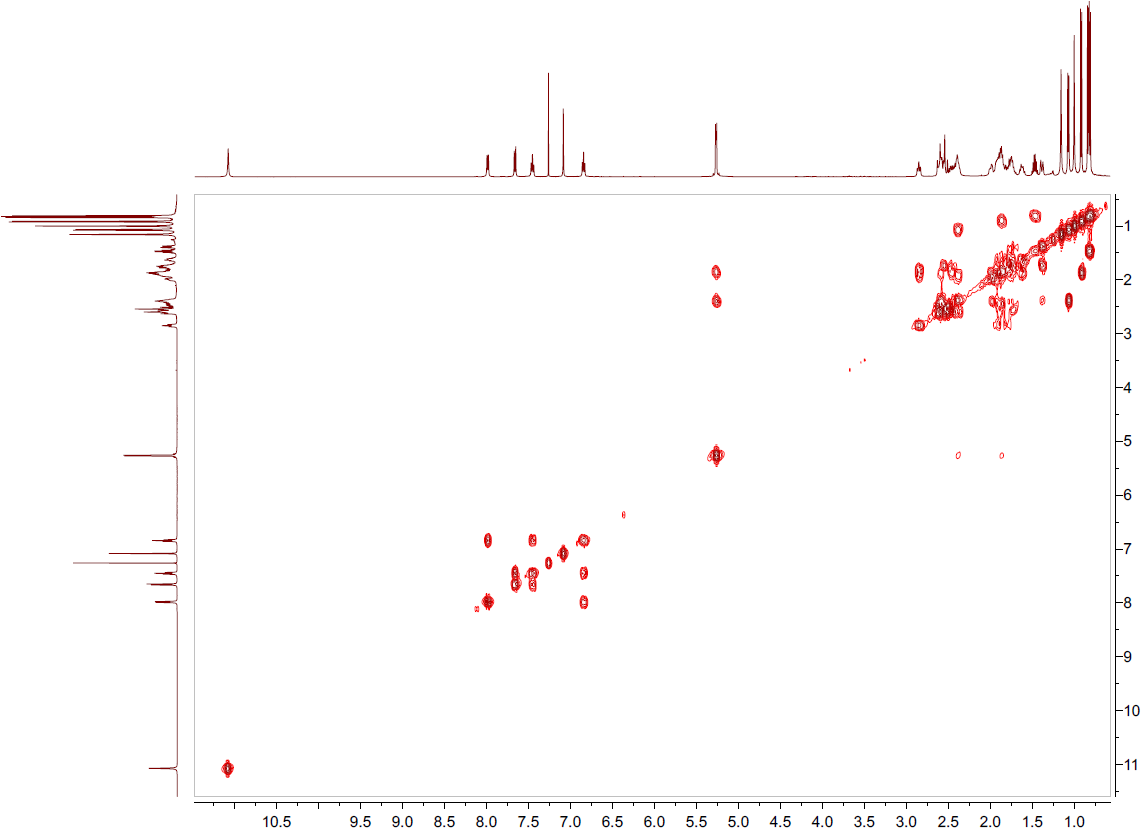


**Figure S9.** COSY spectrum of compound **1** in CDCl_3_.


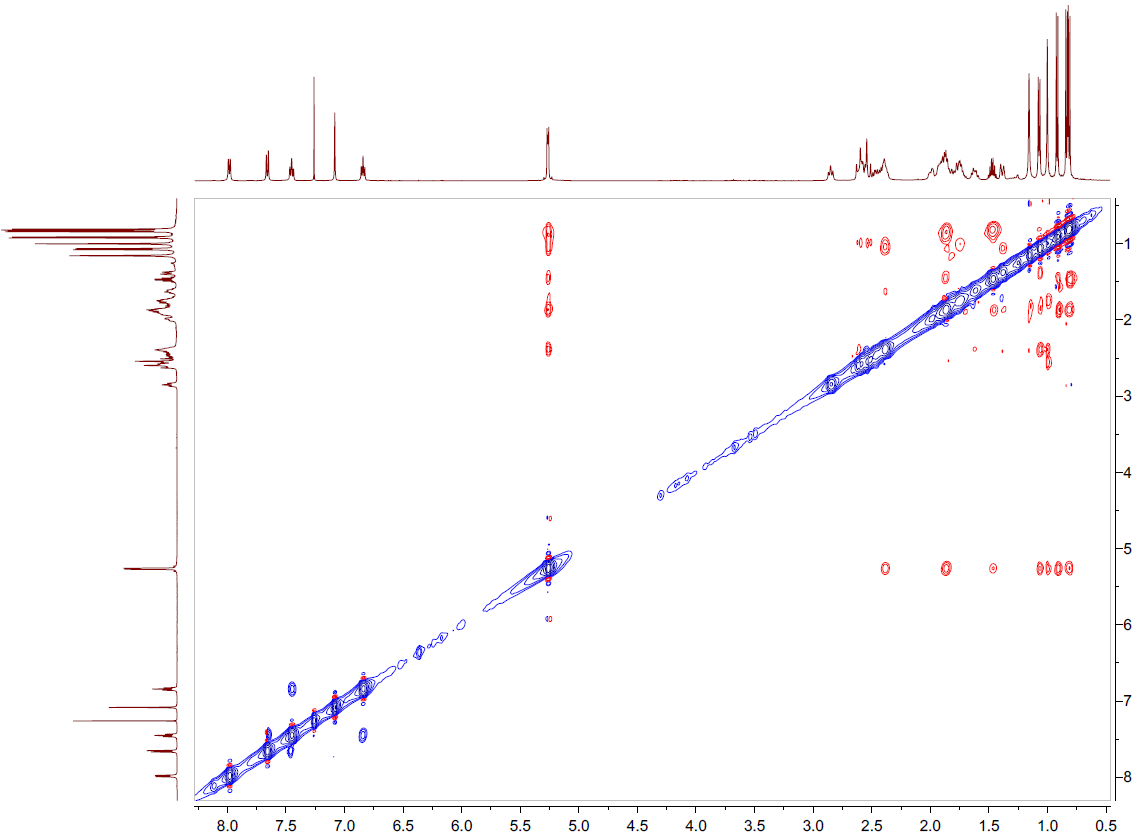


**Figure S10.** NOESY spectrum of compound **1** in CDCl_3_.


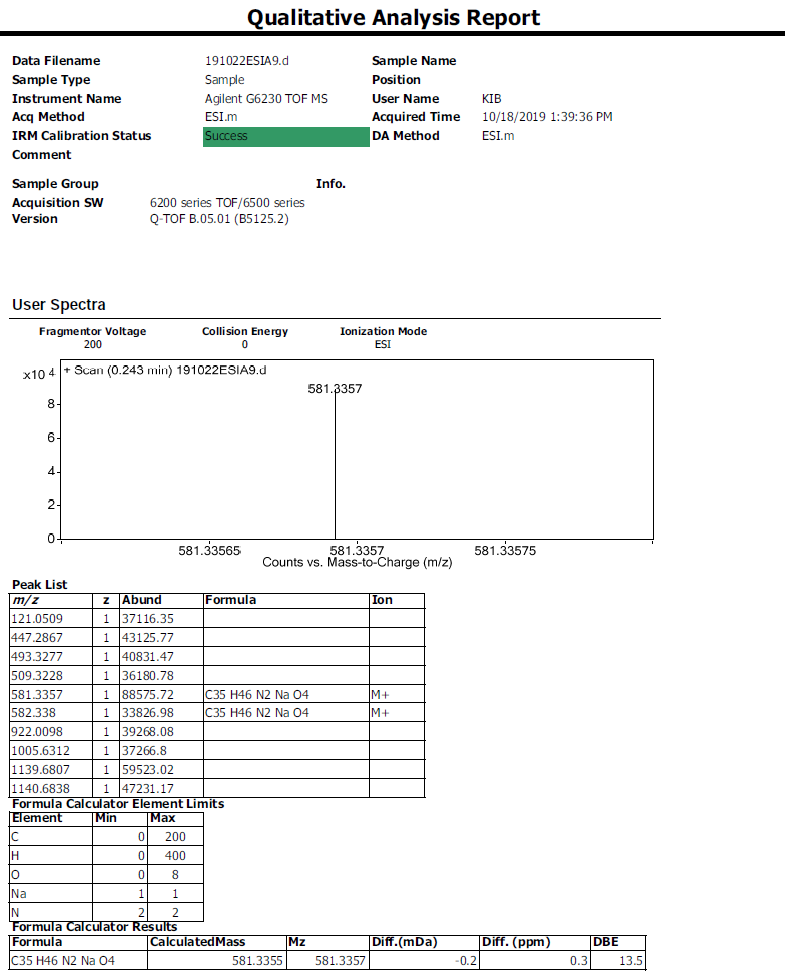


**Figure S11.** HRESIMS spectrum of compound **1**.


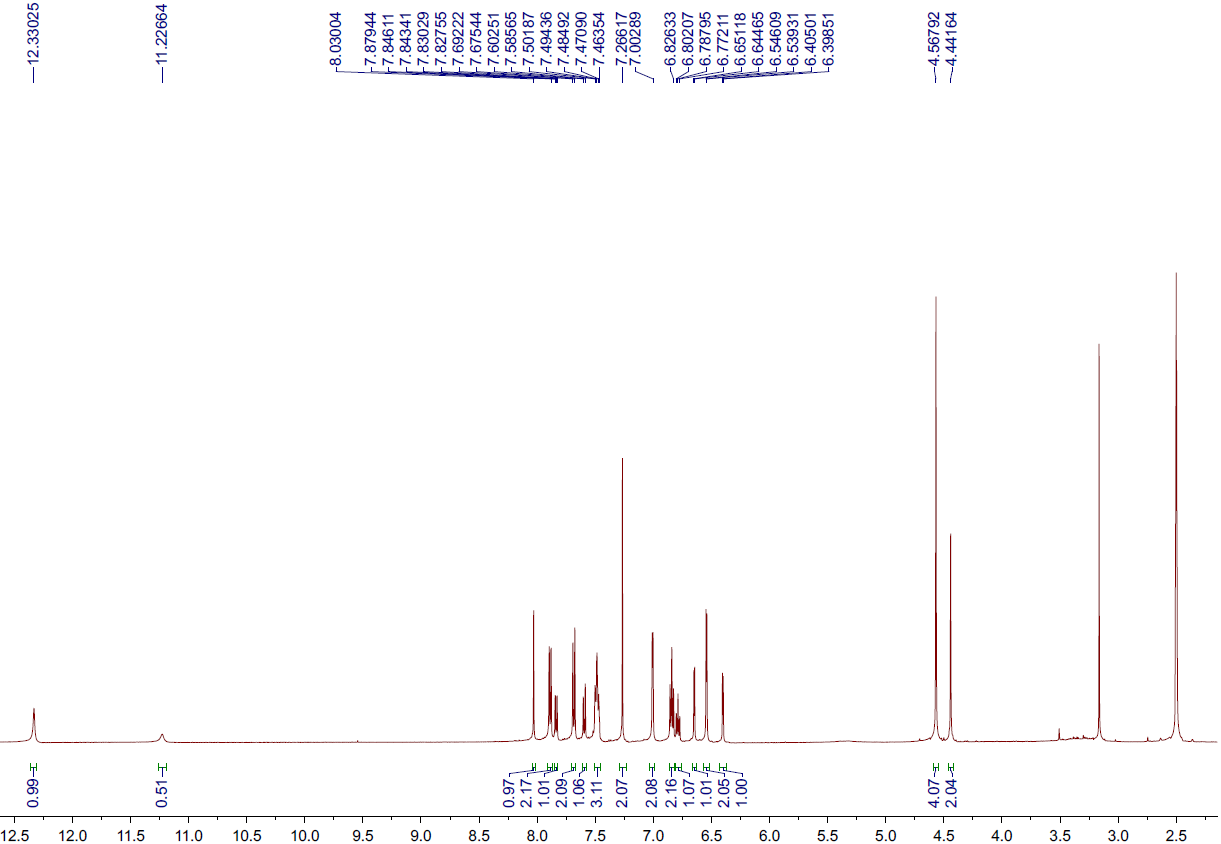


**Figure S12.** ^1^H NMR spectrum of compounds **2** and **3** in DMSO-*d*_6_.


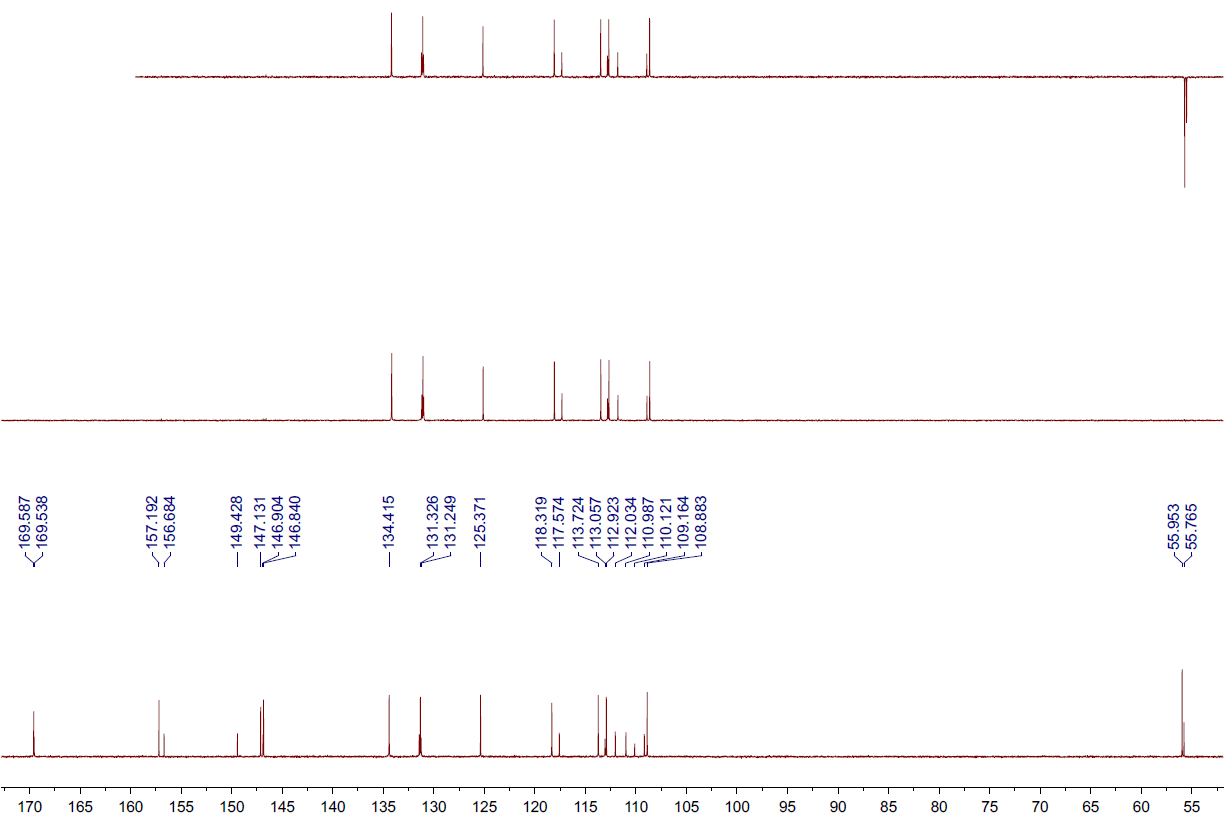


**Figure S13.** ^13^C NMR and DEPT spectra of compounds **2** and **3** in DMSO-*d*_6_.


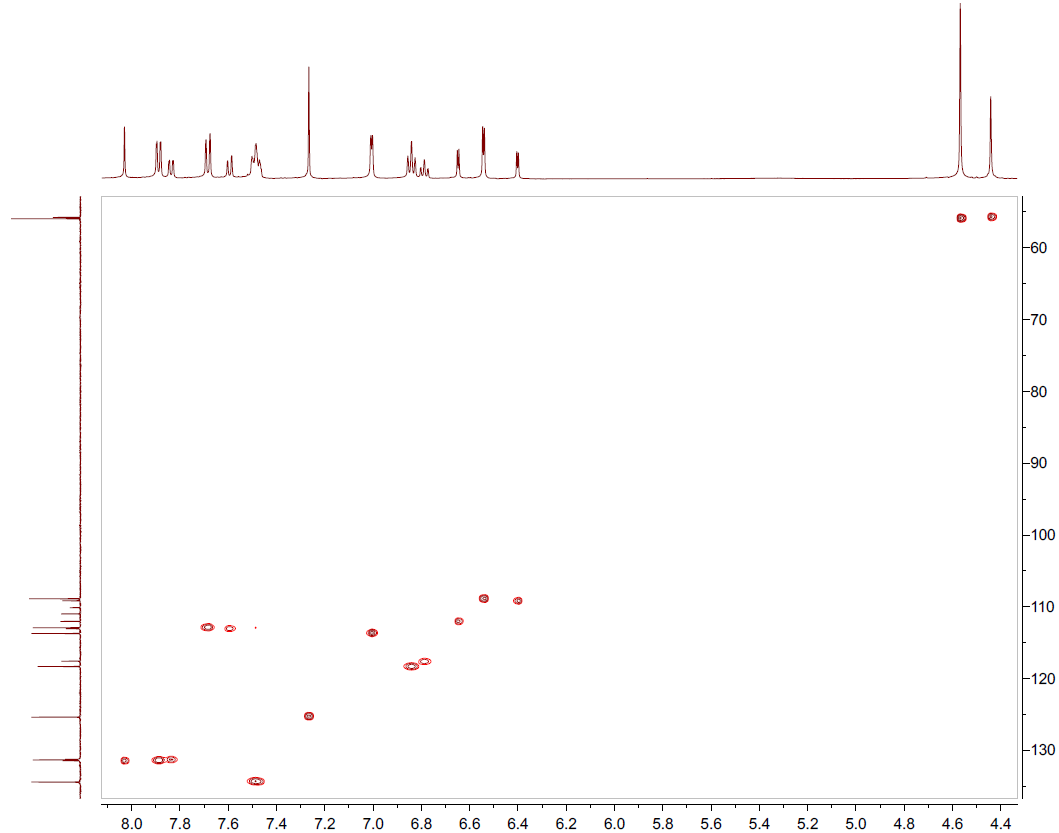


**Figure S14.** HSQC spectrum of compounds **2** and **3** in DMSO-*d*_6_.


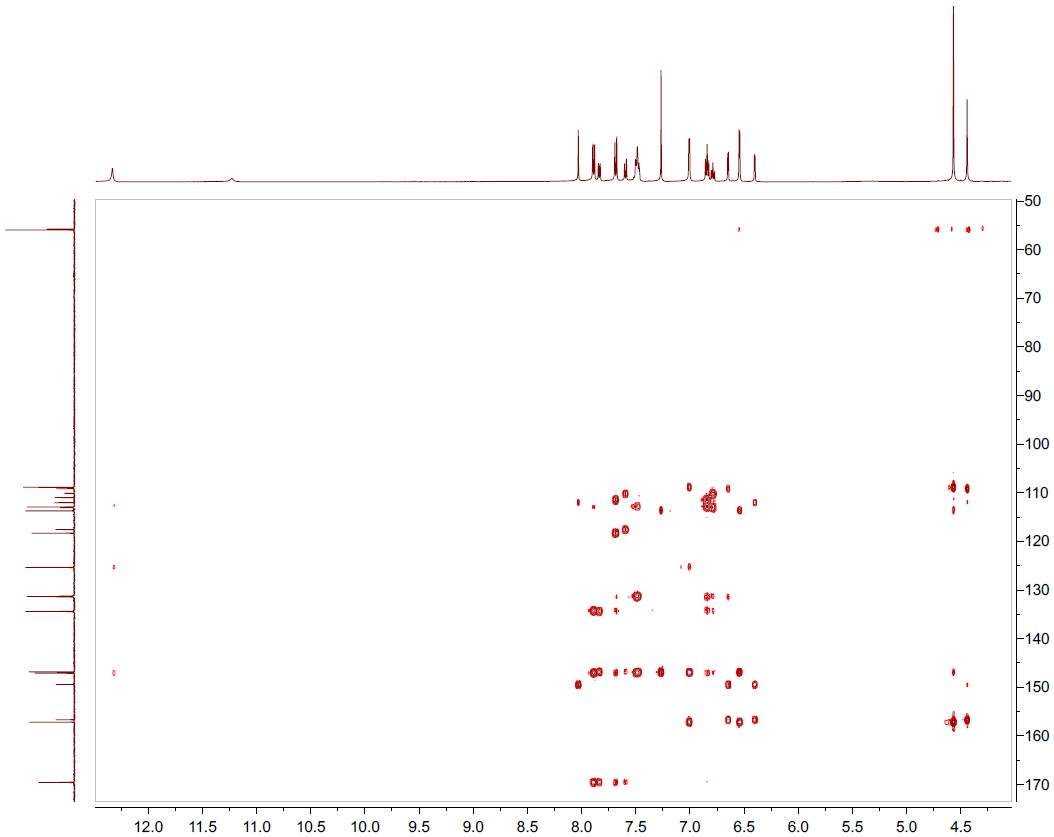


**Figure S15.** HMBC spectrum of compounds **2** and **3** in DMSO-*d*_6_.


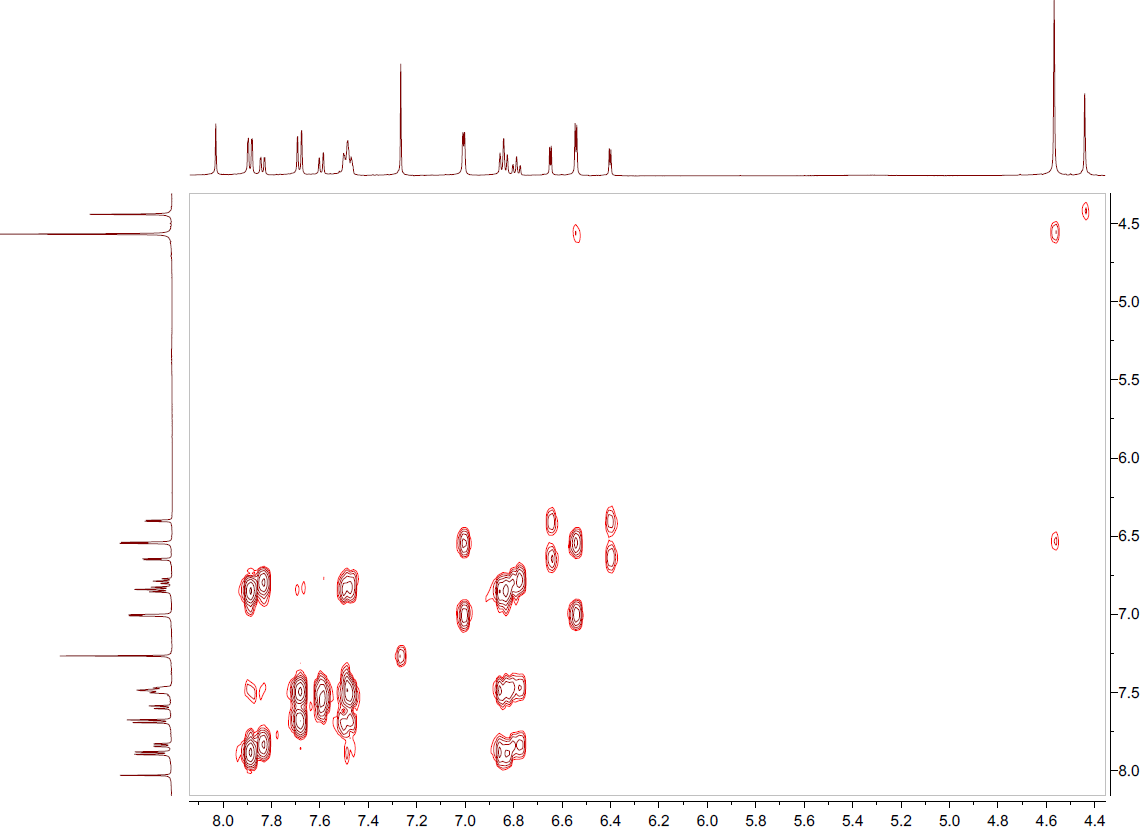


**Figure S16.** COSY spectrum of compounds **2** and **3** in DMSO-*d*_6_.


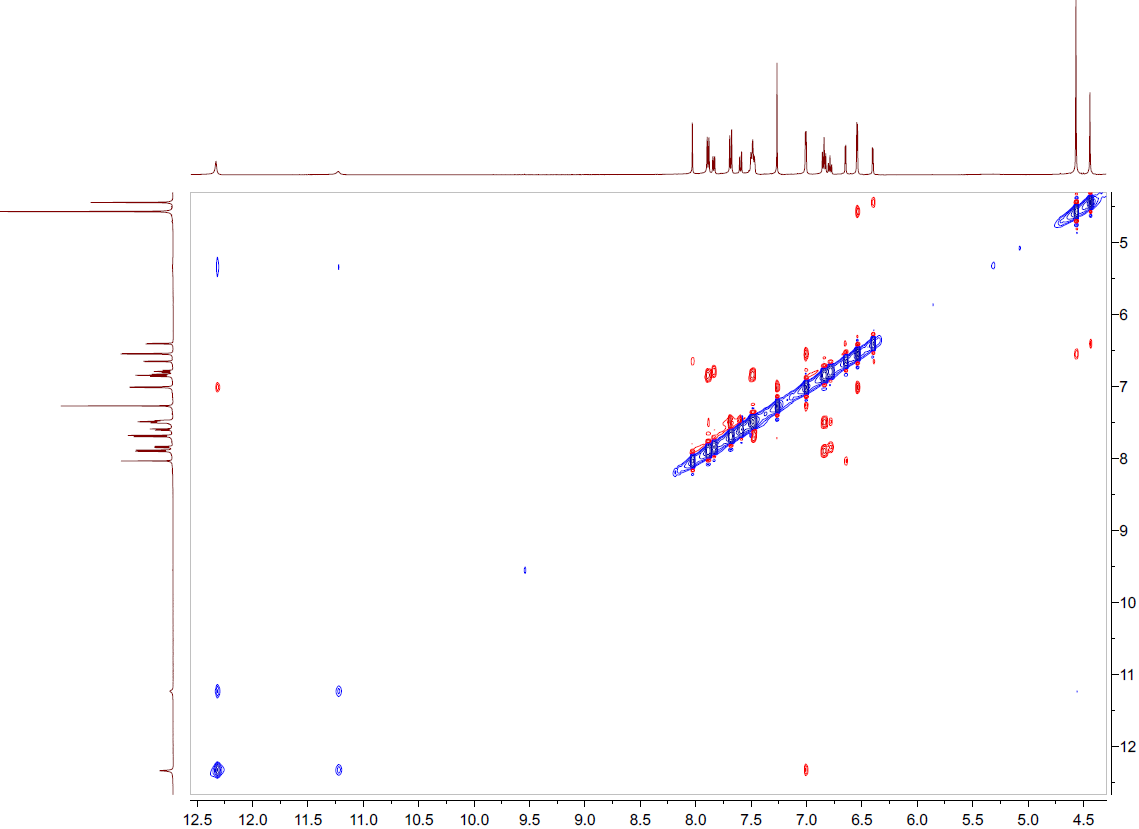


**Figure S17.** NOESY spectrum of compounds **2** and **3** in DMSO-*d*_6_.

**Figure S18.** LC-HRESIMS spectrum of compounds **2** and **3**.


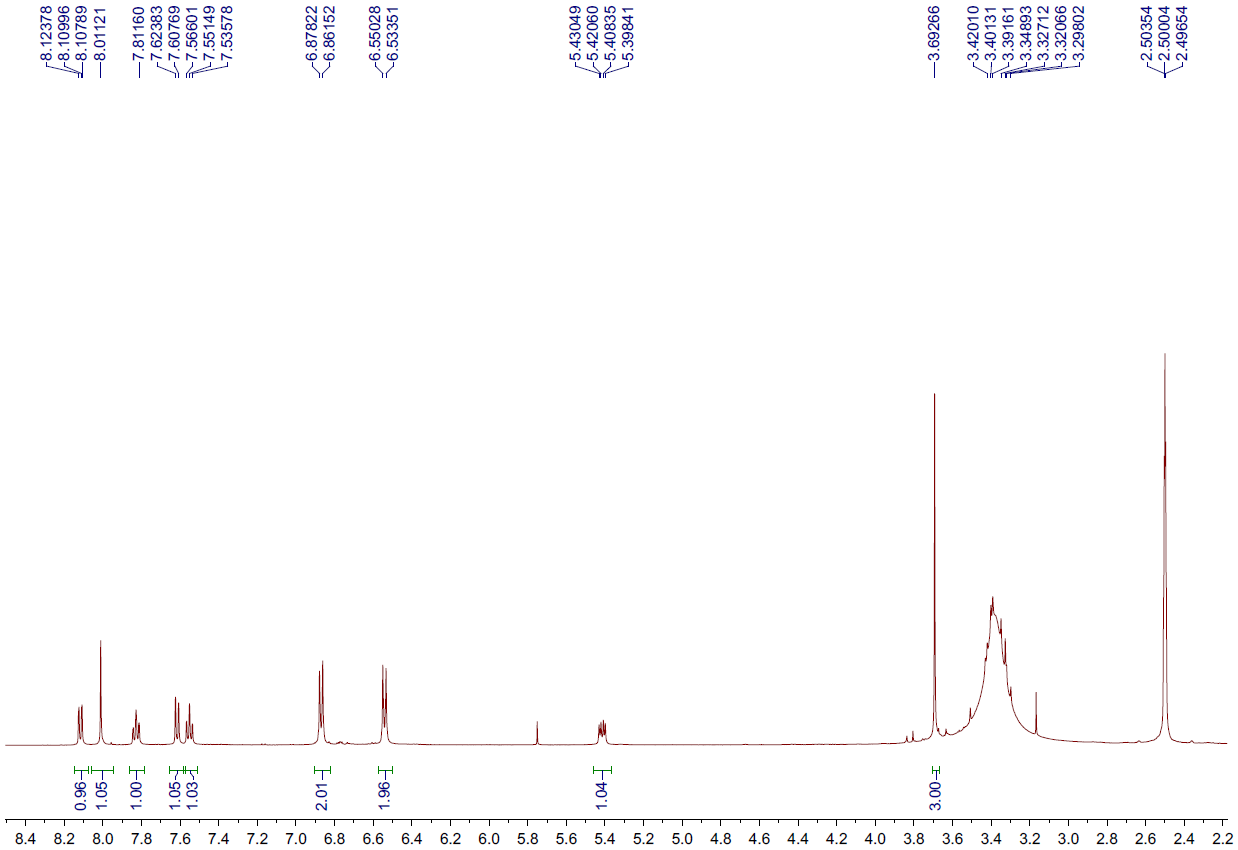


**Figure S19.** ^1^H NMR spectrum of compound compounds **4** and **5** in DMSO-*d*_6_.


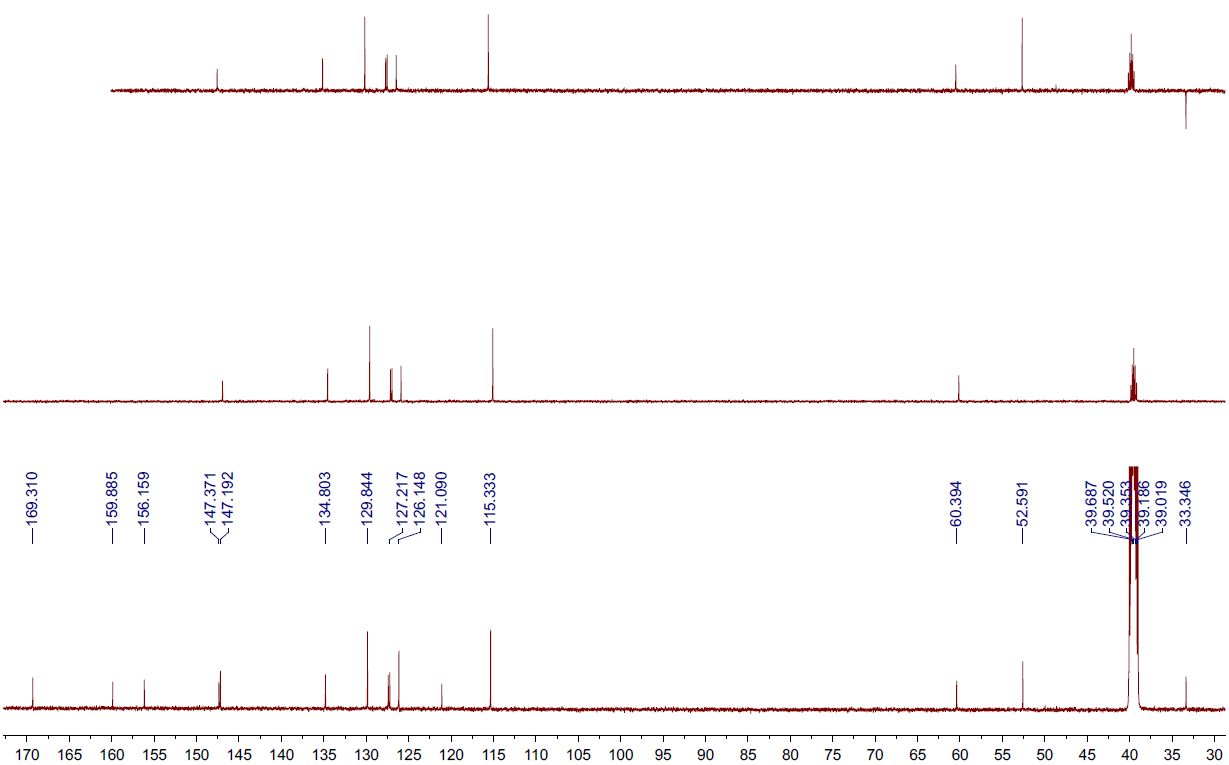


**Figure S20.** ^13^C NMR and DEPT spectra of compounds **4** and **5** in DMSO-*d*_6_.


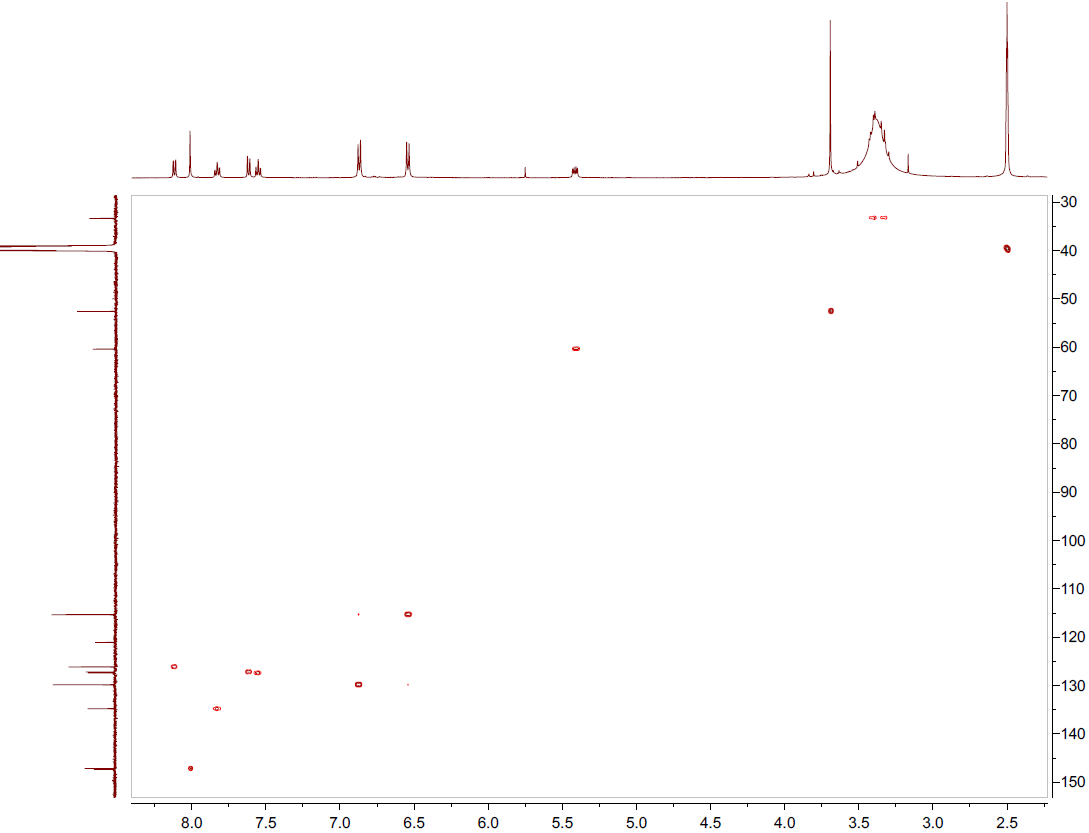


**Figure S21.** HSQC spectrum of compounds **4** and **5** in DMSO-*d*_6_.


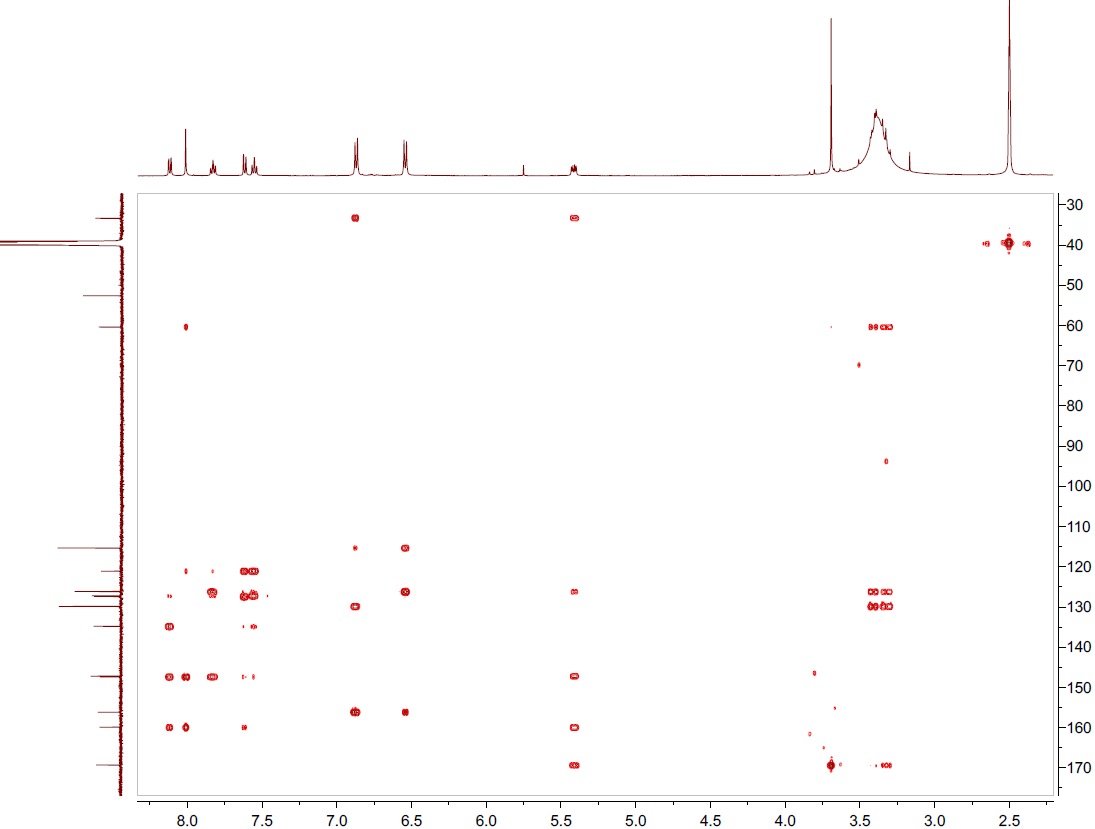


**Figure S22.** HMBC spectrum of compounds **4** and **5** in DMSO-*d*_6_.


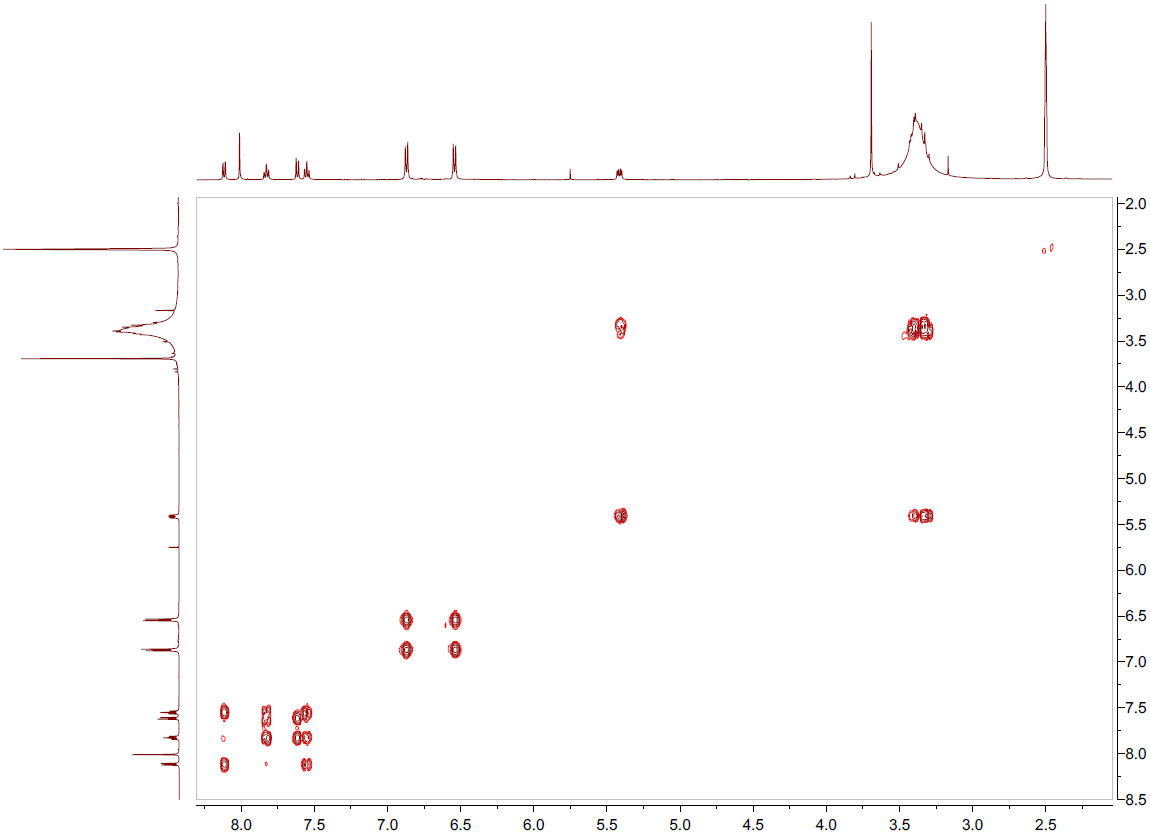


**Figure S23.** COSY spectrum of compounds **4** and **5** in DMSO-*d*_6_.


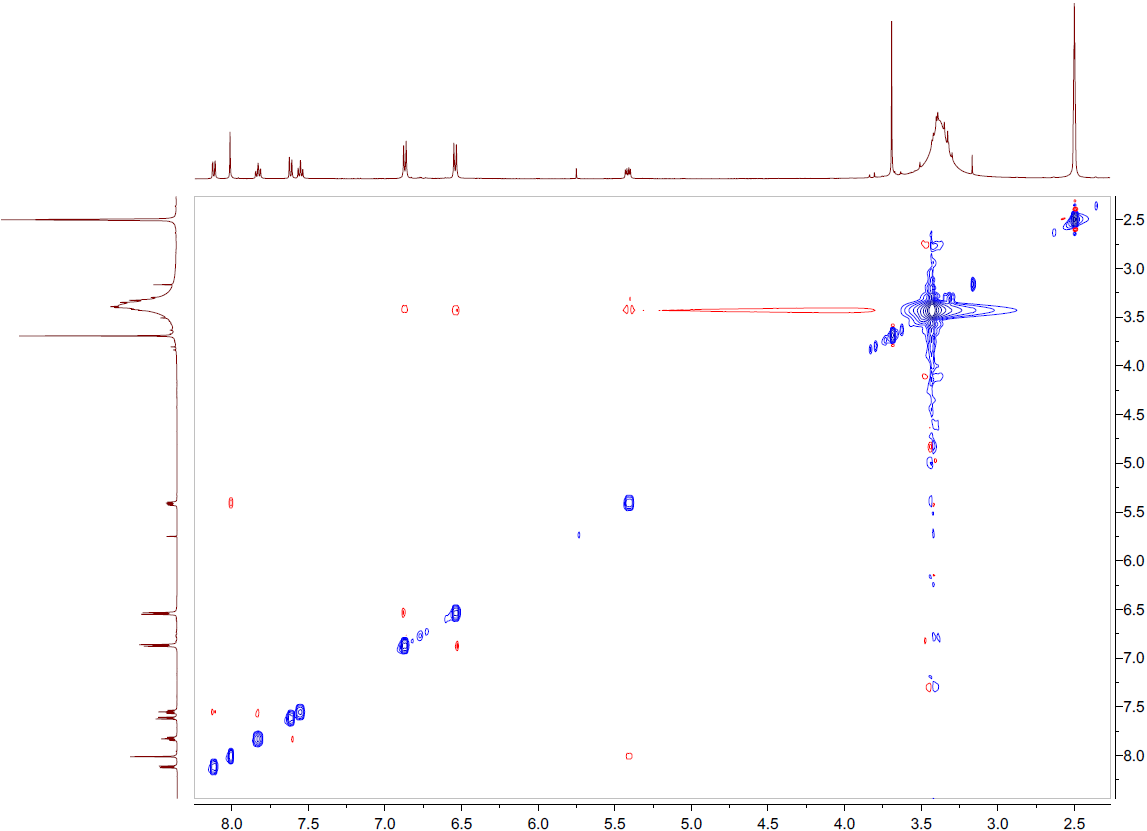


**Figure S24.** NOESY spectrum of compounds **4** and **5** in DMSO-*d*_6_.


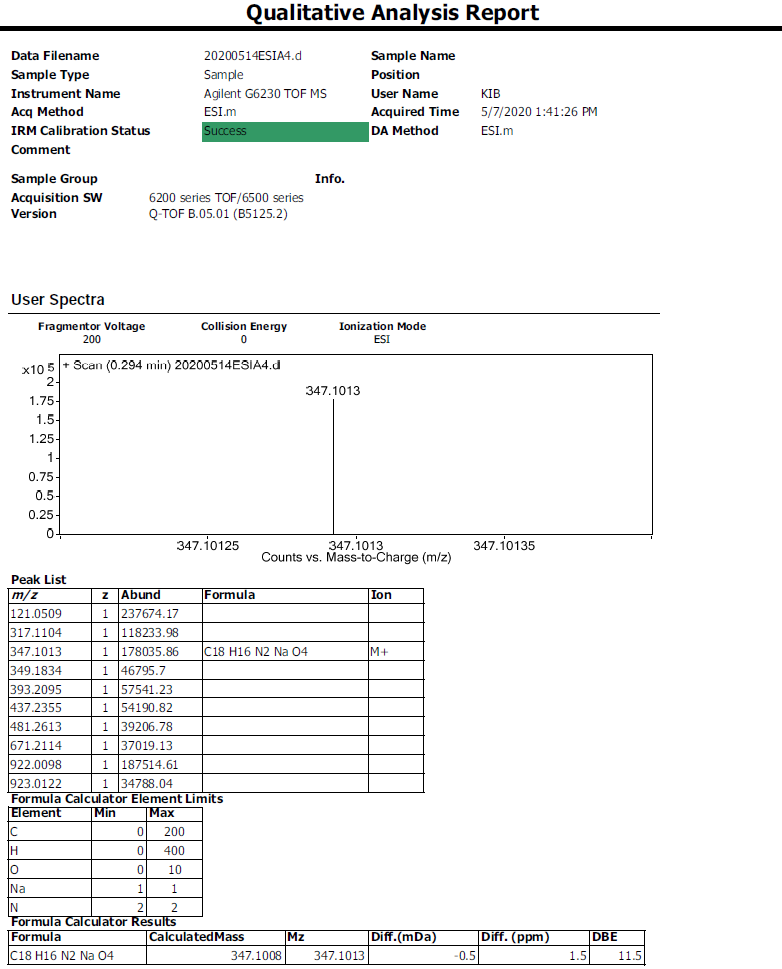


**Figure S25.** HRESIMS spectrum of compounds **4** and **5**.


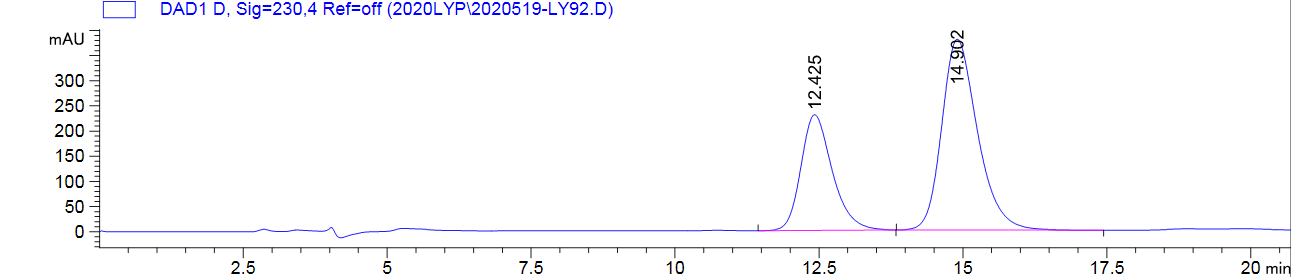


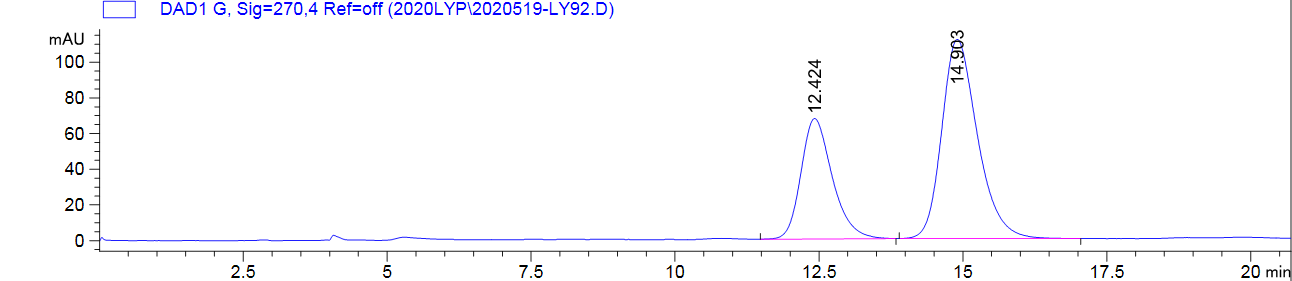


**Figure S26.**Chiral HPLC spectrum of compounds **4** and **5**.
